# Lipid Species in the GI Tract are Increased by the Commensal Fungus *Candida albicans* and Decrease the Virulence of *Clostridioides difficile*

**DOI:** 10.3390/jof6030100

**Published:** 2020-07-03

**Authors:** Jesus A. Romo, Laura Markey, Carol A. Kumamoto

**Affiliations:** 1 Department of Molecular Biology and Microbiology, Tufts University, Boston, MA 02111, USA; 2 Graduate Program in Molecular Microbiology, Graduate School of Biomedical Sciences and Department of Molecular Biology and Microbiology, Tufts University, Boston, MA 02111, USA; Laura.markey@tufts.edu

**Keywords:** *Candida albicans*, *Clostridioides difficile*, colonization, susceptibility, non-esterified fatty acids

## Abstract

Prior antibiotic treatment is a risk factor for *Clostridioides difficile* infection (CDI); the commensal gut microbiota plays a key role in determining host susceptibility to the disease. Previous studies demonstrate that the pre-colonization of mice with a commensal fungus, *Candida albicans*, protects against a lethal challenge with *C. difficile* spores. The results reported here demonstrate that the cecum contents of antibiotic-treated mice with *C. albicans* colonization contained different levels of several lipid species, including non-esterified, unsaturated long-chain fatty acids compared to non-*C. albicans*-colonized mice. Mice fed olive oil for one week and challenged with *C. difficile* spores showed enhanced survival compared to PBS-fed mice. The amount of olive oil administered was not sufficient to cause weight gain or to result in significant changes to the bacterial microbiota, in contrast to the effects of a high-fat diet. Furthermore, the direct exposure of *C. difficile* bacteria in laboratory culture to the unsaturated fatty acid oleic acid, the major fatty acid found in olive oil, reduced the transcription of genes encoding the toxins and reduced the survival of bacteria in the post-exponential phase. Therefore, the effects of *C. albicans* on the metabolite milieu contributed to the attenuation of *C. difficile* virulence.

## 1. Introduction

The opportunistic pathogenic fungus *Candida albicans* is the most common fungal colonizer of the human gastrointestinal tract [1,2,3,4]. *C. albicans* has been shown to affect the host immune system [5,6,7,8,9], and interact with the gut microbiome and pathogenic microorganisms [10,11,12,13,14,15]. Moreover, *C. albicans* has been shown to have significant effects on bacterial population diversity after antibiotic treatment in a murine model [16], suggesting a significant role as a commensal. Currently, there is a lack of knowledge about the role fungi play during the bacterial infection of the gastrointestinal tract. 

*Clostridioides difficile* is an anaerobic, Gram-positive, spore-forming, and toxin-producing bacterial pathogen able to cause mild (antibiotic-associated diarrhea) [17,18] to severe, potentially fatal, gastrointestinal disease (pseudomembranous colitis, toxic megacolon) [19,20,21,22] primarily in those who are elderly, have been hospitalized, and/or received a course of broad-spectrum antibiotics [23,24]. Antibiotic treatment and *Clostridioides difficile* infection (CDI) are inextricably linked and therefore the disease has been labeled as an urgent public health concern by the United States White House’s National Action Plan for Combating Antibiotic-resistant Bacteria (CDC.gov) due to its high morbidity, the medical costs it creates [25,26,27], and the treatment difficulty (i.e., the emergence of drug resistance to fluoroquinolones and disease relapse) [20,28,29,30,31,32,33,34,35,36]. 

*C. difficile* spores are acquired via the fecal–oral route and germinate in the intestinal tract. Antibiotic treatment leads to the disruption and decrease in diversity of the protective host gastrointestinal microbiota, allowing *C. difficile* to flourish, colonize the lower gastrointestinal tract, secrete toxins, and cause epithelial damage [37]. *C. difficile* infection (CDI) is a complex disease whose pathology is primarily caused by the secretion of the toxins TcdA and TcdB. Both toxins are monoglycosyltransferases that target Rho family GTPases leading to cell cytoskeleton disruption and cell death [38].

Host susceptibility and CDI severity is influenced by the host bile acid metabolome. Host-derived primary bile acids such as cholate (CA), deoxycholate (DCA), and taurocholate (TCA) are able to stimulate *C. difficile* spore germination [39,40]. Once these primary bile acids enter the small and large intestines, they can be converted into a variety of chemically diverse species of secondary bile acids by the resident microbiota [41]. Secondary bile acids inhibit *C. difficile* spore germination and are therefore crucial for protectiveness [23,39,42,43,44]. Buffie and co-workers identified key bacterial taxa required for *C. difficile* colonization resistance [43]. Among these, *Clostridium scindens* was highly associated with CDI resistance. Furthermore, the removal of *C. scindens* from the murine gastrointestinal tract by antibiotic treatment led to a reduction in secondary bile acids and increased sensitivity to CDI, while the administration of *C. scindens* restored secondary bile acid pools and CDI resistance. Thus, the alteration of microbiota composition and the associated changes in the bile acid metabolome accompanied changes in susceptibility to CDI. These studies highlight the role of the host microbiota in creating a metabolic environment that is unfavorable to *C. difficile*. Additionally, the host diet is able to influence CDI susceptibility. Recently, Hryckowian and co-workers demonstrated that the supplementation of microbiota accessible carbohydrates (MACs) into the diet of antibiotic-treated mice reduced *C. difficile* burdens while murine diets deficient in MACs perpetuate CDI [45]. Moreover, MACs diet selected specific microbial taxa whose metabolic end products consist of high levels of short-chain fatty acids (SCFA) such as acetate, propionate, and butyrate, which are known to have antagonistic effects on *C. difficile*. This study showed an association between the host diet, levels of SCFA, and the *C. difficile* burden. Interestingly, this reduction in virulence was independent of the toxin production as the authors identified high levels of toxin.

The role of host bacterial microbiota during CDI and in preventing CDI has been well characterized, but a role for fungal gastrointestinal colonizers has not been well studied and is therefore largely unknown. Previous clinical observations have yielded contradicting reports suggesting that the fungus *C. albicans* appears to have a protective [46,47] effect or play a negative [48,49] role by exacerbating CDI or reducing the effectiveness of FMT [50]. Our group recently utilized a murine model of the *C. albicans* gastrointestinal colonization followed by CDI in order to investigate the role of fungi [51]. In these experiments, antibiotic-treated mice were pre-colonized with *C. albicans* for prolonged periods of time (3 weeks). Long-term colonization allowed the host gastrointestinal microbiota to begin to recover from the antibiotic treatment in the presence of *C. albicans*. Mice were then administered clindamycin and challenged with a highly virulent *C. difficile* strain (UK1) [44]. Mice pre-colonized with *C. albicans* displayed increased survival showing that the *C. albicans* pre-colonization was protective in this model.

In this study, we further characterized the ecological effects of *C. albicans* that contribute to the protective effects we observed previously [51]. We hypothesized that *C. albicans* modifies the metabolite milieu of the gastrointestinal tract environment, which could influence the ability of *C. difficile* to cause disease. We performed an untargeted metabolomics analysis of cecum contents of antibiotic-treated mice with or without *C. albicans* colonization and found that *C. albicans* pre-colonized mice had increased levels of non-esterified fatty acids (NEFA), particularly unsaturated fatty acids. To mimic this increase in fatty acids, mice were fed olive oil, an oil rich in triglycerides containing an unsaturated fatty acid, oleic acid. These mice displayed an increased survival rate when challenged with CDI. Moreover, the oleic acid supplementation of *C. difficile* cultures led to a reduction in the transcription of genes encoding *C. difficile* toxins. These findings are consistent with the model in which the environmental modification of the host gastrointestinal tract by *C. albicans* includes increasing levels of unsaturated fatty acids, which play a role in the overall reduction of the virulence of *C. difficile*.

## 2. Materials and Methods

### 2.1. Strains and Growth Conditions

*C. albicans* strain CKY101, [52] a virulent strain derived from the sequenced strain SC5314, was used for all studies. Cells were prepared for mouse inoculation by growth at 37 °C in YPD broth (1% yeast extract (BD, Sparks, MD, USA cat. 212750), 2% peptone (Difco, Detroit, MI, USA cat. 0118-17-0), and 2% glucose (Sigma-Aldrich, St. Louis, MO, USA cat. G8270)) [53] for 21–24 hrs.

*C. difficile* strain UK1, a NAP1/027/BI human epidemic strain [44], was used for all studies. Cultures were grown in pre-reduced TY broth (3% tryptose, 2% yeast extract, 0.1% thioglycolate, pH 7.4) [54]. Spores were isolated as previously described [39] except that the gradient purification was omitted. For the enumeration of *C. difficile* vegetative cells and spores in the extracts from mice, the samples were plated on pre-reduced TCCFA plates (Taurocholate (Calbiochem, San Diego, CA, USA cat. 580217), cycloserine (Sigma-Aldrich, St. Louis, MO, USA cat. C6880), cefoxitin (Sigma-Aldrich, St. Louis, MO, USA cat. C4786), fructose (Macron Fine Chemicals, Center Valley, PA, USA cat. 7756-12)) [40] and incubated at 37 °C for 2 days in an anaerobic chamber. Spores were enumerated by heating the samples at 60 °C for 10 min, followed by plating on pre-reduced TCCFA plates.

### 2.2. GI Colonization in Mice

All the experiments using animals were done in compliance with the NIH Guide for the Care and Use of Laboratory Animals and Tufts University IACUC guidelines. Animal experimentation was approved by the Tufts Institutional Animal Care & Use Committee, 10/08/2018, protocol number B2018-84.

Five-week-old female C57BL/6 mice (Jackson Laboratory, Bar Harbor, ME, USA) were co-housed in a large cage (24” × 17”) for a week, and then treated with the antibiotic cefoperazone (Sigma-Aldrich, St. Louis, MO, USA cat. C4292, 0.5 gm/L) in drinking water for 10 days while in the large cage. All the mice were tested at the end of cefoperazone treatment and shown to be negative for cultivable fungi on YPD-SA agar medium (YPD agar plus 100 μg/mL streptomycin (Sigma-Aldrich, St. Louis, MO, USA cat. S6501) and 50 μg/mL ampicillin (Sigma-Aldrich, St. Louis, MO, USA cat. A9518)) incubated for 2 days at 37 °C. On the 10th day of antibiotic exposure, some mice were inoculated orally with 5 × 10^7^ *C. albicans* cells in 25 μL. 

All mice were transferred from the large cage to standard sized cages, housing 4 mice per cage and switched to water without cefoperazone. *C. albicans* colonization was measured as described previously [51]. Briefly, 3–4 fecal pellets were collected from each animal into a pre-weighed tube containing PBS. The tube was weighed after collection. Pellets were then homogenized using a microtube vortex, serially diluted, and plated for CFU on YPD-SA. 

After 14 days of resting, some mice without *C. albicans* inoculation were given 3.3 µL olive oil (Sigma-Aldrich, St. Louis, MO, USA cat. O1514) (3 mg) plus 15 µL PBS by gently pipetting into their mouths. Control mice received 15 µL PBS. These administrations were given daily for the remainder of the experiment.

Mice were transferred to 2 mice/cage, 17 days post-cefoperazone. 20 days post-cefoperazone treatment, and they were inoculated intraperitoneally with clindamycin (Sigma-Aldrich, St. Louis, MO, USA cat. C5269) (10 mg/kg). On the next day, the mice were orally inoculated with *C. difficile* spores (3–5 × 10^5^ spores per mouse). All the mice used in these experiments were shown to be negative for *C. difficile* colonization prior to inoculation with *C. difficile* spores by collecting fecal pellets and plating on pre-reduced TCCFA.

The mice were weighed daily at the same time of day and sacrificed 5 days post-inoculation or when moribund. If the mice exhibited severe signs of illness (extreme inactivity, hunched posture, ruffled fur), they were considered moribund and were sacrificed. The mice were also sacrificed if their weight loss exceeded 20%. Within one experimental trial (i.e., one batch of mice), some of the mice were sacrificed on day 2 post-inoculation for various analyses while the other mice were monitored for survival over 5 days. The survival data show the combined results of the mice from multiple experimental trials.

The relative weights were compared using the *t* test. Survival was compared using the log rank test. Samples of cecum contents were plated on pre-reduced TCCFA to enumerate *C. difficile*.

### 2.3. C. difficile Toxin Titer Assay

African green monkey kidney epithelial cells (Vero cells) (ATCC CCL-81) were seeded in 96-well microtiter plates at a concentration of 4.3 × 10^3^ cells per well in DMEM (Corning Cell Gro, Corning, NY, USA cat. MT10-013CV) with 10% heat-inactivated fetal bovine serum (Atlanta Biologicals S11150) and 1% MEM non-essential amino acids (ThermoFisher Scientific, Waltham, MA, USA cat. 11140076) and allowed to adhere for 24 h at 37 °C and 5% CO_2_ as previously described [51,55]. The cecum contents were weighed and diluted with 10 times the volume of DMEM with 10% FBS and 1% MEM non-essential amino acids. Serial three-fold dilutions were made in DMEM with 10% FBS and 1% MEM non-essential amino acids, added to adhered Vero cells, incubated for 24 h at 37 °C in 5% CO_2_ and scored visually at 10× magnification for cell rounding. The toxin titer is defined as the inverse of the greatest dilution that resulted in 100% cell rounding.

### 2.4. Histology

Cecum tissue from the mice sacrificed two days post-inoculation with *C. difficile* was fixed in buffered formalin and processed for staining with Hematoxylin and Eosin (H&E) by the Tufts Comparative Pathology Core facility. Scoring was conducted by a blinded investigator on 30–40 10× fields of view for each tissue. The scores were acquired by dividing the number of fields showing infiltration/inflammation by the total number of fields for that tissue. Each point on the graph represents an individual mouse. A non-parametric t-test was used to compare the groups.

### 2.5. Metabolomic Analysis of Murine Cecum Contents

Cecum contents from the antibiotic-treated mice with or without *C. albicans* pre-colonization were collected, rapidly frozen in a dry ice/ethanol bath and stored at −80 °C. Samples were shipped to Metabolon (Durham, NC, USA), for metabolite extraction and untargeted metabolomic analysis as described by Metabolon. Briefly, the proteins were precipitated with methanol under vigorous shaking for 2 min followed by centrifugation. The resulting extract was analyzed using 4 ultra-high-performance LC–MS/MS methods. All methods utilized Waters ACQUITY ultra-high-performance liquid chromatography (UHPLC) and a Thermo Scientific Q-Exactive high resolution/accurate mass spectrometer interfaced with a heated electrospray ionization (HESI-II) source and an Orbitrap mass analyzer operated at 35,000 mass resolution. The MS analysis alternated between the MS and data-dependent MS^n^ scans using dynamic exclusion. The scan range varied slightly between the methods but covered 70–1000 *m/z*. Raw data were extracted, peak-identified and QC processed using Metabolon’s hardware and software. Mass spectrometry data were analyzed using MetaboAnalyst 4.0 (University of Alberta, Edmonton, AB, Canada) [56,57].

### 2.6. Microbiota Analysis

The distal portion of the cecum and its contents were dissected from the mice two days post-inoculation with *C. difficile*. The distal tip was dissected and rapidly frozen on dry ice. Microbial DNA was extracted using the QIaAMP PowerFecalPro DNA Kit (Qiagen, Hilden, Germany cat. 12830-50) according to the manufacturer’s protocols including the recommended additional steps to ensure the optimal yield of DNA from Gram-positive bacteria. The libraries were prepared from each sample and sequenced as described [58]. Briefly, the PCR amplification of the V4 region of the 16S rRNA gene was performed with 515F and 806R primers that included adapters for Illumina sequencing and 12-mer Golay barcodes to allow for multiplexing. Two hundred and fifty bp paired-end sequencing was performed using an Illumina MiSeq according to the manufacturer’s protocols. Base calling was performed using CASAVA 1.8 and the resulting fastq files were used for downstream analysis using QIIME 2 (2018.8) [59]. Raw sequences were demultiplexed and filtered using the q2-demux plugin followed by DADA2 [60] for denoising (q2-dada2). Amplicon sequences were aligned using mafft [61] (q2-alignment). Phylogeny trees were constructed using the de novo phylogenetic tree from fasttree2 [62] (q2-phylogeny). Alpha-diversity metrics (Shannon, Chao1, and Simpson) and Beta-diversity (weighted UniFrac [63]) were estimated using the q2-diversity. Principal coordinate analysis (PCoA) was used to summarize the weighted UniFrac distance matrix. PERMANOVA analysis was performed using QIIME2. The operational taxonomic units (OTUs) were determined by aligning reads to the Greengenes Database (version 13_8) at 99% identity [64]. Feature tables describing the relative abundance of bacterial taxa were used for the analysis of diversity within each sample.

The total levels of bacteria per cecal tip sample were measured by qPCR using eubacterial primers (Supplementary Table S1). The qPCR reactions were conducted using SYBR Green PCR Master Mix (Applied Biosystems, Beverly, MA, USA cat. 4364346) and a LightCycler 480 II (Roche, Basel, Switzerland) instrument. The normalized abundance of bacterial genera was calculated by multiplying the fraction of total reads for a genus by the total level of bacteria per mg of cecum sample (in arbitrary units).

### 2.7. Cytokine Gene Transcription

For the measurement of cytokine gene transcription, the mice were sacrificed prior to or two days post-inoculation with *C. difficile* and the colon tissue was frozen in RNALater (Invitrogen, Carlsbad, CA, USA cat. AM7021) at −80 °C. RNA was purified with Trizol (Invitrogen, Carlsbad, CA, USA cat 15596026) extraction and column purification, using the Ambion Purelink RNA mini kit (Invitrogen, Carlsbad, CA, USA cat 12183018A). DNA was eliminated with on-column DNase treatment. cDNA preparation with Superscript III (Invitrogen, Carlsbad, CA, USA cat 18080051) was performed using the manufacturer’s protocol. The qRT-PCR reactions were performed as described above for qPCR. The triplicate samples were measured; the controls lacking a template did not yield products. Standard curves were generated and all the results were normalized to the level of GAPDH transcription in each sample. The primers are listed in Supplementary Table S1.

### 2.8. C. difficile Gene Transcription

For the growth curves of the *C. difficile* cells in the presence or absence of oleic acid diluted in ethanol, TY broth was used. Overnight cultures were diluted to an OD_600_ of 0.02 into pre-reduced TY broth containing 0.05 mM oleic acid, 0.6 mM oleic acid or the vehicle (ethanol). The addition of oleic acid did not result in a change in the pH of the broth. The cultures were incubated at 37 °C in an anaerobic chamber. The samples were taken at various times to measure the OD_600_ or plate for CFU from vegetative cells and spores using pre-reduced BHIS plates (3.7% brain heart infusion extract, 0.5% yeast extract, 0.1% L-cysteine, 1.5% agar [65]). The samples were taken at 8 h or 24 h, mixed with RNAprotect, centrifuged at 21,000 g for 10 min at 4 °C and the cell pellets were frozen at −80 °C.

For the analysis of epithelium-associated bacteria, the mice were sacrificed two days post-inoculation with *C. difficile* spores. The ceca were removed, the cecum wall was washed twice in RNAprotect and the epithelium was scraped into RNAprotect using a glass slide. This sample was centrifuged at 4000 g for 7 min and the pellets were frozen at −80 °C. 

The RNA was extracted from these samples by bead beating in Trizol with 0.1 mm zirconia/glass beads, 3 × 1 min in a Biospec Bead Beater 24, followed by purification using the PureLink mini RNA extraction kit. The cDNA was prepared with random priming and MultiScribe Reverse Transcriptase using the Applied Biosystems High Capacity cDNA synthesis kit. The primers were designed using Primer-BLAST to identify the primers predicted to lack targets in other bacteria from the Refseq representative genomes database. The PCR products were sequenced to verify the specificity of the primers. 

Gene transcription was measured by quantitative RT PCR using an Applied Biosystems StepOne System. The transcription of *rpoA* was used to normalize the transcription of the genes of interest. Primers failed to yield products when the cDNA derived from uninfected cecum epithelium, extracted and processed as above, was used as the template (Supplementary Figure S1). 

To increase the ability to detect the *C. difficile* gene transcription in samples scraped from infected murine ceca, a pre-amplification step was included. A single primer specific for the *C. difficile* gene of interest and upstream of the primer pair used for PCR amplification was designed using Primer-BLAST. Since all cDNA molecules are copied from the positive, sense (mRNA) strand, they represent the negative strand. Therefore, the upstream primer was a forward primer and would prime from the cDNA copies. Twelve cycles of conventional PCR were performed using a mix of single gene specific primers. The pre-amplified cDNA was then used as template for qRT PCR. The experiment shown in Supplementary Figure S2 shows that the samples retained their quantitative relationship and that gene transcription was detected with a higher sensitivity after pre-amplification. All *C. difficile* genes other than *tcdB* and *tcdA* required pre-amplification to be detected in the samples of scraped epithelia.

### 2.9. Statistical Analysis

Statistical analyses were performed using GraphPad Prism (San Diego, CA, USA), Excel or Metaboanalyst (University of Alberta, Edmonton, AB, Canada).

## 3. Results

### 3.1. Altered Levels of Unsaturated Fatty Acids and Other Lipid Species in the Cecum Contents of C. albicans-Colonized Mice

**Figure 1:**
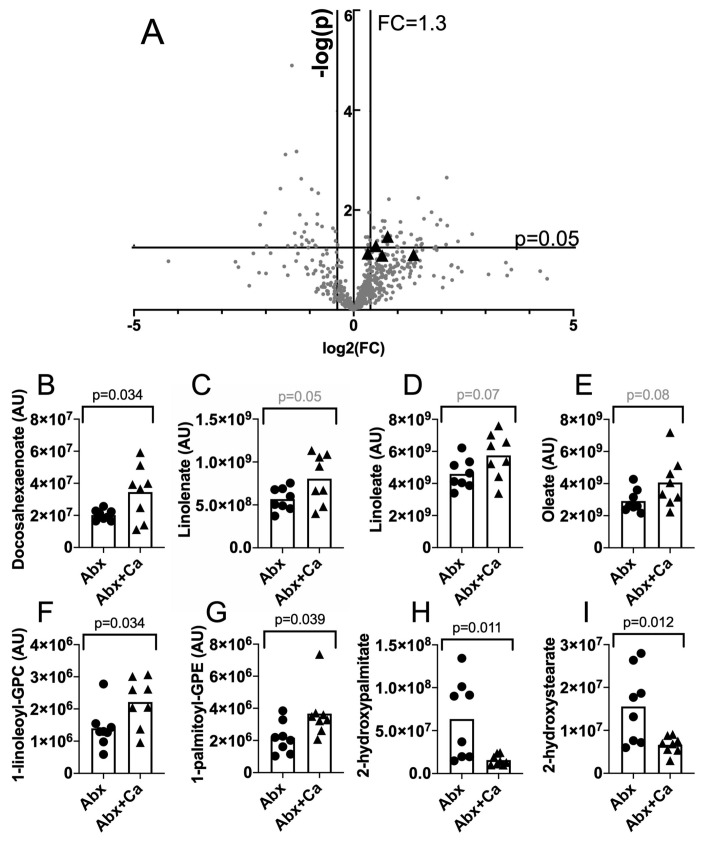
Untargeted metabolomic analysis of the cecum contents reveals higher levels of compounds related to linolenic acid metabolism in *C. albicans*-colonized mice. Mice were treated with antibiotics as described in Materials and Methods (cefoperazone, 10 days, rest 20 days, clindamycin ip). On the day following clindamycin treatment, the mice were sacrificed and the contents of the cecum were collected and frozen rapidly in dry ice/ethanol. These samples were analyzed by Metabolon using LC–MS/MS. Results were analyzed using Metaboanalyst. (**A**) Of the 595 compounds identified by Metaboanalyst, 590 are shown in a volcano plot (log2(FC) versus -log10(*p*)). The black triangles show the compounds in the enriched, linoleic and linolenic acid metabolism pathway. (**B**–**I**) Compounds showing altered levels or a trend toward altered levels in *C. albicans*-colonized mice. Each symbol shows the results from an individual animal. Bar shows the mean. Compounds (B–D) are in the linoleic and linolenic acid metabolism pathway. Circles, antibiotic treated, uncolonized; triangles, antibiotic treated, *C. albicans* colonized.

**Figure 2:**
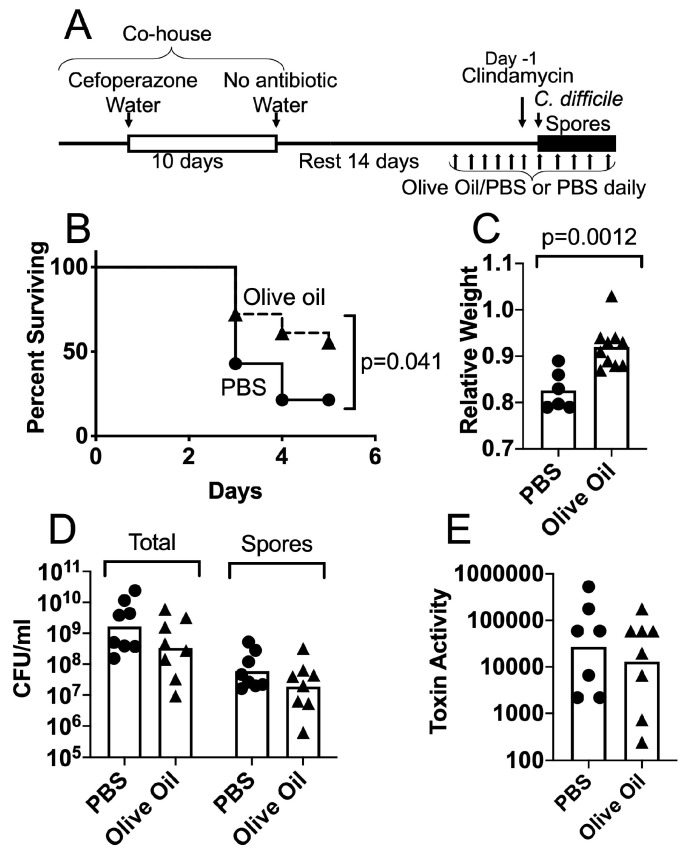
Olive oil feeding enhances the survival of the challenge with *C. difficile* spores in the antibiotic-treated mice. Outline of the mouse regimen is shown in (**A**). The C57BL/6 mice were co-housed in a large cage and received antibiotics as described in the Materials and Methods (cefoperazone 10 days). Mice were then transferred to the standard size cages with water without additives, rested 14 days, and then received a daily oral administration of olive oil with PBS or PBS only. Mice received clindamycin ip and the following day received *C. difficile* spores (3–5 × 10^5^ spores of strain UK1, a NAP1/027/BI human epidemic strain) by oral inoculation. (**B**) Survival of the mice was monitored for 5 days post-inoculation with spores (PBS, *n* = 14; Olive oil, *n* = 18; log rank test *p* = 0.041). (**C**) Panel shows the relative weights of the surviving mice on day 3 post-inoculation (*t* test *p* = 0.0012). (**D**) *C. difficile* bacteria were enumerated by plating the homogenized cecum contents collected from the mice sacrificed on day 2 post-inoculation. Homogenates were plated on a TCCFA medium (total) or heated at 60 °C for 10 min and plated (spores). (**E**) *C. difficile* toxin activity in the cecum contents from the mice sacrificed on day 2 post-inoculation was measured using a cell-rounding assay. The inverse of the greatest dilution that yielded 100% cell rounding is plotted. Circles, antibiotic treated, uncolonized; triangles, antibiotic treated, *C. albicans* colonized.

To determine the effect of *C. albicans* colonization on the metabolite milieu of the intestinal tract, mice were treated with antibiotic to increase their susceptibility to CDI, but instead of receiving spores, the mice were sacrificed and the contents of the cecum were collected and rapidly frozen. The antibiotic regiment consisted of 10 days of exposure to cefoperazone in drinking water while being co-housed, followed by oral inoculation with *C. albicans* (or not) as described below (Figure 2A). The mice were then rested for 20 days in smaller groups (4/cage with *C. albicans*-colonized mice separated from the non-colonized mice) with water lacking antibiotics. Fecal pellets collected during the resting period were plated to detect *C. albicans* CFU. All the mice were positive for *C. albicans* on day 6 or day 17 post-inoculation ((Supplementary Figure S3) consistent with the previous demonstrations of colonization in this model [16,51]. Mice were given clindamycin (10 mg/kg body weight) by intraperitoneal injection and sacrificed on the following day. Eight mice per group, taken from two different experimental trials, were analyzed. The cecum contents were analyzed using LC–MS/MS by Metabolon as described and the results were analyzed using Metaboanalyst [56,57] (Figure 1A). Of the 595 total compounds analyzed, 44 showed a fold change ≥ 1.3 (colonized/uncolonized mouse ceca) and a *p* ≤ 0.05 (Supplementary Table S2). 

To identify the compounds from pathways enriched in ceca of *C. albicans*-colonized mice, we used the pathway enrichment tool within MetaboAnalyst. Analyzing only statistically significantly different compounds revealed no known pathways, but including compounds that showed a trend towards significance (*p* < 0.1) allowed the detection of one pathway: five compounds in the linoleic (C18:2) and linolenic (C18:3) acid metabolism pathway (Figure 1A, black triangles, Figure 1B) were present at higher levels in *C. albicans*-colonized mice. All three 18-carbon non-esterified unsaturated fatty acids showed trends toward higher levels in *C. albicans*-colonized mice (Figure 1C–E). Furthermore, the increase in lyso-phospholipids (Figure 1F,G) could indicate a hydrolysis of phospholipids and the release of non-esterified fatty acids while the decrease in hydroxy fatty acids (Figure 1H,I) could indicate a reduced conversion of fatty acids into other forms. Therefore, the results showed altered levels of lipid compounds, including non-esterified, unsaturated fatty acids, in the cecum contents of *C. albicans*-colonized mice.

### 3.2. Protective Effects of Olive Oil Feeding on Antibiotic-Treated Mice Challenged with C. difficile Spores

We proposed that the levels of lipid compounds in the GI tract influence the course of CDI in antibiotic-treated mice. To initiate the test of this hypothesis, we analyzed the effects of increasing levels of unsaturated fatty acids and other lipid compounds through feeding. The mice were treated with cefoperazone and clindamycin as described above and shown in Figure 2A. Importantly, no mice were given *C. albicans* for this set of experiments. After two weeks of resting post-cefoperazone treatment, the mice were orally administered a modest amount of olive oil daily for 1 week prior to the *C. difficile* challenge and continuing to the end of the experiment. The timeline of this experiment is shown in Figure 2A. In a pilot experiment, the mice received either olive oil (composed predominantly of triglycerides containing oleic acid (C18:1)) or fish oil (source of long-chain poly-unsaturated fatty acids such as DHA), orally with PBS (Supplementary Table S3). The results of this pilot experiment suggested that larger amounts of both oils had some effect and olive oil had a slightly stronger effect. Although fish oil contains long-chain poly-unsaturated fatty acids, some of its components have anti-inflammatory effects and fish oil administration is sometimes protective and sometimes not protective against infectious challenge [66]. Olive oil, in contrast, is composed primarily of triglycerides containing oleic acid (C18:1), a compound that showed a trend towards higher levels in the ceca of *C. albicans*-colonized mice (Figure 1E).

Further studies of olive oil administration showed that this treatment had a protective effect. The mice were treated as illustrated in Figure 2A and were fed either 3.3 µL olive oil with PBS, or PBS alone. This treatment did not lead to significant weight gain in the olive oil-fed mice (Supplementary Figure S4). After the administration of clindamycin (10 mg/kg ip), the mice were challenged with 3–5 × 10^5^ spores of *C. difficile* strain UK1, a NAP1/027/BI human epidemic strain, and weight loss and survival were monitored for 5 days post-inoculation. As shown in Figure 2B, the mice receiving olive oil treatment showed evidence of improved survival relative to the mice receiving PBS alone. Of the 18 mice that were fed with olive oil, eight died, none were sacrificed when moribund or due to weight loss, and 10 survived. Of the 14 mice that did not receive olive oil, seven died, four were sacrificed due to weight loss, none were sacrificed when moribund and three survived. The survival curves were significantly different (*p* = 0.041, Kaplan–Meier log rank test). The relative weights of mice were determined on the days following the *C. difficile* challenge (Supplementary Figure S5). The relative weights of the surviving mice on day 3 post-challenge with spores were significantly different, with the oil-fed mice showing a higher weight, consistent with the enhanced resistance to CDI (Figure 2C, *t* test *p* = 0.0012). 

To analyze the disease parameters, the mice were treated as shown in Figure 2A and then sacrificed two days post-inoculation with spores. The total *C. difficile* CFU/gm cecum contents or spore CFU/gm cecum contents did not differ significantly between the mice with or without olive oil treatment (Figure 2D). The toxin activity measured in cecum contents did not differ significantly between the two groups of mice (Figure 2E). 

**Figure 3:**
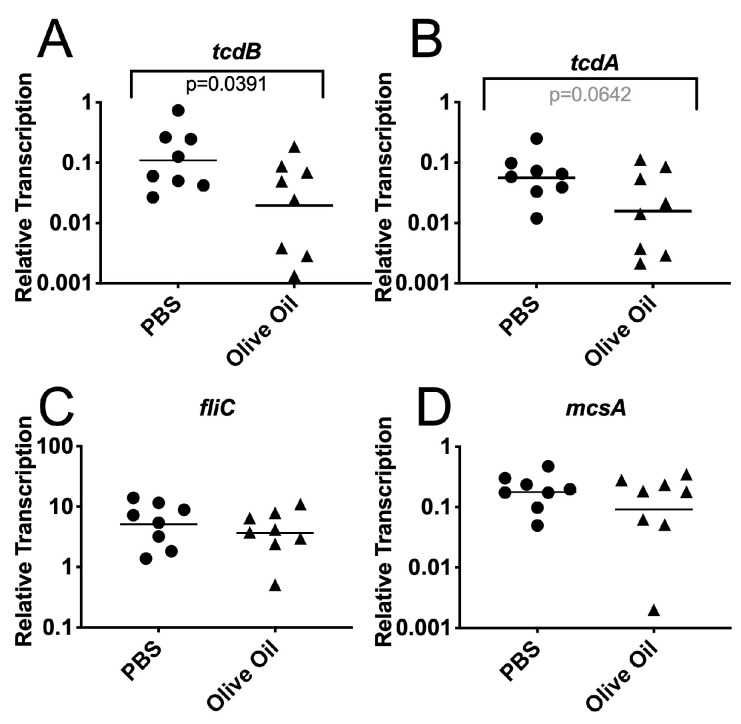
Reduced transcription of *tcdB* in the epithelium-associated *C. difficile* cells harvested from the olive oil-fed mice. Mice were treated as described in Figure 2A. On day 2 post-inoculation with spores, the mice were sacrificed and their cecum was removed. The cecum was opened, washed with RNAprotect and the wall was scraped into RNAprotect to recover the epithelium and enrich for associated bacteria. RNA was extracted and the gene transcription measured in cDNA using qRT PCR. Transcription of (**A**) *tcdB*, (**B**) *tcdA*, (**C**) *fliC*, and (**D**) *mcsA* was normalized using *rpoA* transcription. Circles, antibiotic treated, uncolonized; triangles, antibiotic treated, *C. albicans* colonized.

We also analyzed the *C. difficile* gene transcription in the bacterial cells recovered from infected mice. The mice were treated as shown in Figure 2A and sacrificed two days post-challenge with spores. The ceca were dissected, opened and washed twice in RNA protect. The washed cecum wall was then scraped into RNAprotect to recover the epithelium and enrich for epithelium-associated bacteria. The RNA was extracted from the samples and the *C. difficile* gene transcription was measured using RT PCR as described in the Materials and Methods. The *C. difficile* toxin genes *tcdB* and *tcdA* were transcribed at high enough levels to be detected in these mixed samples. Other *C. difficile* genes were poorly expressed and a pre-amplification method was used to increase the sensitivity of detection. This pre-amplification consisted of 12 rounds of PCR using only one sense strand primer and increased the sensitivity while retaining the quantitative assessment of gene transcription (Supplementary Figure S2). The transcription of the toxin gene *tcdB* was significantly lower in epithelium-associated *C. difficile* cells from olive oil-fed mice (Figure 3A, *t* test *p* = 0.0391). The transcription of *tcdA* showed a trend towards lower transcription in olive oil-fed mice (*t* test *p* = 0.0642). Other genes such as *fliC* or *mcsA* (CDR20291_0013) did not show significant differences in their transcription. These results indicate that although the overall toxin levels in the cecum contents were not significantly different, the possible ability to produce toxin (specifically TcdB) by *C. difficile* cells in the vicinity of the epithelium was affected by the treatment with olive oil. These results are consistent with the observation that olive oil feeding reduced the virulence of *C. difficile*, but this reduction in virulence is most likely multifactorial.

Histological analysis and the scoring of cecum and colon tissue from olive oil-fed or control mice sacrificed two days post-inoculation with spores showed regions of infiltration and edema, consistent with the damage due to *C. difficile* infection and no overall difference between the two treatment groups (Supplementary Figure S6). 

**Figure 4:**
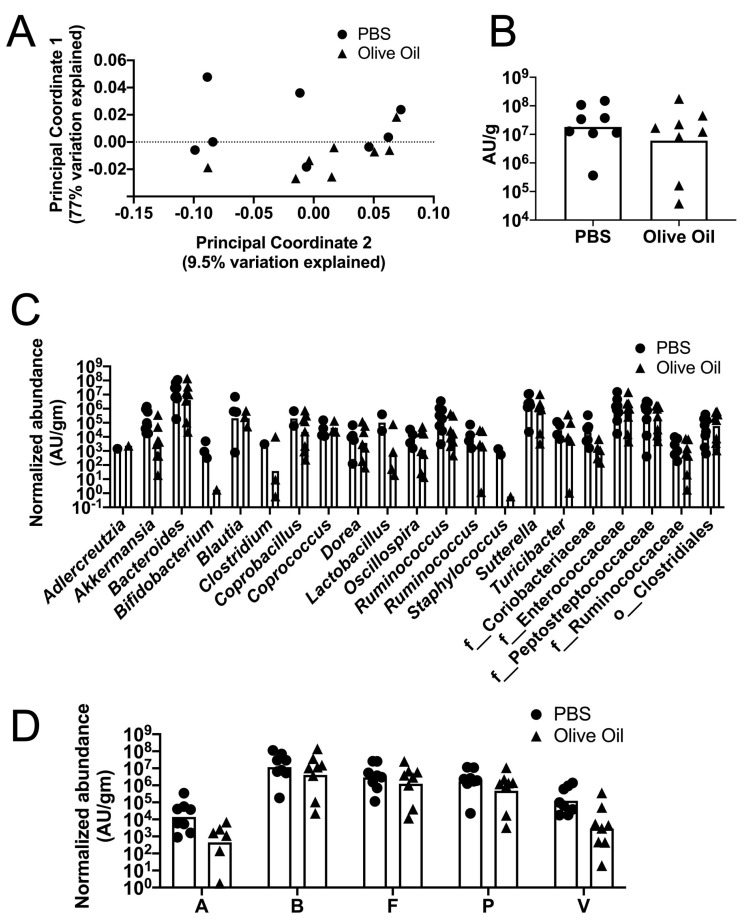
Analyses of the cecal bacterial communities in the PBS or olive oil-fed mice infected with *C. difficile*. Mice were treated as described in Figure 2A. On day 2 post-inoculation with *C. difficile* spores, the mice were sacrificed and their ceca were removed. Bacterial composition was analyzed as described in the Materials and Methods with QIIME 2. (**A**) Weighted UniFrac distances were used to perform a Principal coordinate analysis. (**B**) Total levels of bacteria per cecal tip sample were measured by qPCR using eubacterial primers and normalized to milligrams of cecum sample used for DNA extraction. Bar indicates geometric mean. (**C**) Normalized abundance per mg of cecum sample for the bacterial genera in the cecal microbiota (*n* = 8 mice per group). (**D**) Phyla are abbreviated as follows P = Proteobacteria; V = Verrucomicrobia; A = Actinobacteria; F = Firmicutes; B = Bacteroidetes. The black circles indicate the PBS-treated, *C. difficile*-challenged mice. The black triangles indicate the olive oil-fed, *C. difficile*-challenged mice.

The cecal microbiota of the mice treated with cefoperazone followed by clindamycin and then given olive oil or PBS and finally challenged with *C. difficile* spores was analyzed (Figure 4). The mice were sacrificed two days post-inoculation with spores and the cecum tip was harvested; DNA was extracted as described in the Materials and Methods. The bacterial community composition was characterized by sequencing the V4 region of the 16S rRNA gene and analyzed using QIIME 2 (2018.8) [59] as described in the Materials and Methods. The principal coordinate analysis (PCoA) of the weighted UniFrac distance for the bacterial communities in the PBS versus the olive oil mouse ceca treatment is shown in Figure 4A. The PERMANOVA analysis of these results did not detect a statistically significant difference between the treatment groups (*p* = 0.311). The total levels of bacteria were measured using qPCR and universal 16S rRNA primers (Supplementary Table S1). The total number of bacteria detected per milligram of cecum sample was not significantly different between the mice that received PBS versus the mice that received olive oil (Figure 4B). 

To test for possible differences in the specific genera within the microbiota between the PBS and olive oil-fed groups, we compared the abundance of bacterial genera, normalized to the total level of bacteria per milligram of cecum sample. Normalization was used to account for the differences in the total levels of bacteria per mg of sample. All the bacterial genera with a median fraction greater than 0 in at least one of the two groups were included in order to identify the consistently observed genera (Figure 4C). These mice had received multiple antibiotic treatments and were infected with *C. difficile*, and they were colonized with relatively few genera. We detected a total of 44 genera and 16 exhibited a median greater than 0 in at least one group. The normalized abundance of the genera was compared between the PBS or the olive oil-fed mice using an unpaired t-test. None of the genera identified displayed significant differences. Additionally, we did not detect any significant differences in the phyla composition between the groups (Figure 4D).

To detect a possible effect of olive oil on the relative abundance of rare taxa, we measured the community diversity using the Chao1 estimator (Supplementary Figure S7A). The microbiota did not exhibit increased diversity between the PBS or olive oil treatment when analyzed using the Chao1 or other measures (e.g., Simpson index and Shannon index, Supplementary Figure S7B,C). The above results demonstrate that the concentration of olive oil supplementation did not have significant effects on the host microbiota composition and diversity analyzed at the genus level.

**Figure 5:**
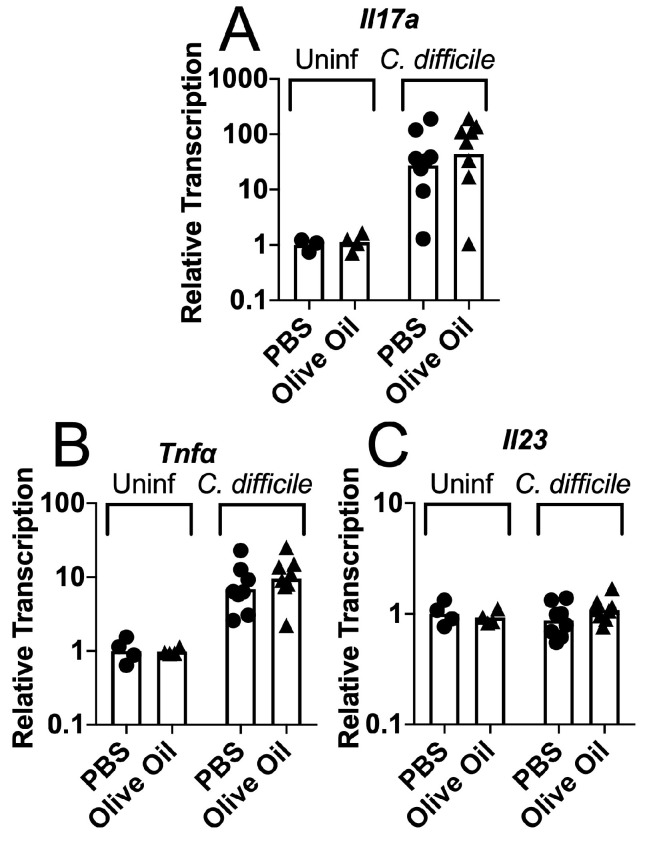
Cytokine gene transcription in the *C. difficile*-challenged, olive oil-fed mice. Mice were treated as described in Figure 2A. Before or on day 2 post-inoculation with spores, the mice were sacrificed and their colons were removed and stored in RNALater. RNA was extracted and the gene transcription was measured in cDNA by qRT PCR. Transcription of (**A**) *Il-17a*, (**B**) *Tnfα*, and (**C**) *Il-23* was normalized to transcription of *Gapdh*. Circles, antibiotic treated, uncolonized; triangles, antibiotic treated, *C. albicans* colonized.

Pre-colonization with the commensal fungus *C. albicans* resulted in higher levels of transcription of the cytokine gene *Il17a* in colon tissue harvested from the antibiotic-treated, *C. difficile*-challenged mice [51]. To determine whether the olive oil administration similarly altered the host cytokine response to *C. difficile* spore challenge, we analyzed the cytokine gene transcription in colon tissue taken either before or 2 days after inoculation. RNA was extracted from these tissues and gene transcription was measured in cDNA using quantitative real-time PCR (qRT PCR). Differences between the olive oil-fed and the PBS-fed mice in the transcription of genes encoding IL-17A, IL-23 and TNF-α were not detected either before or after spore challenge (Figure 5). Transcription of *Il17a* and *Tnfα* increased in response to the *C. difficile* challenge. 

In summary, we observed the reduced virulence of *C. difficile* in antibiotic-treated mice fed olive oil and a reduction in the toxin gene transcription in the epithelium-associated *C. difficile* bacteria. Changes in the composition of bacterial microbiota or the cytokine responses of the host were not detected. These results are consistent with the model in which changes in the metabolome due to *C. albicans* colonization or olive oil feeding can ameliorate the course of CDI.

### 3.3. Effects of Oleic Acid on C. difficile Growth and Toxin Gene Transcription

These results showed that the olive oil treatment did not exert a strong effect on the GI tract environment (bacterial microbiota, cytokine responses), raising the possibility that the treatment had a direct effect on *C. difficile* bacteria. Olive oil is primarily composed of triglycerides of oleic acid, which are hydrolyzed in the upper GI tract. We therefore examined whether non-esterified oleic acid had a direct effect on the physiology of *C. difficile*. 

**Figure 6:**
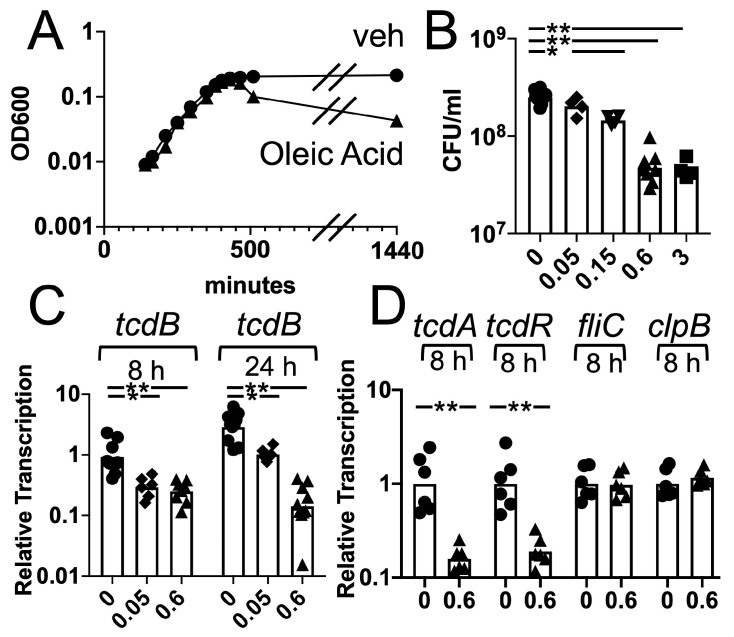
Altered survival and gene transcription in the *C. difficile* cells grown in the presence of oleic acid. *C. difficile* cells of the strain UK1 were grown overnight in a pre-reduced TY (3% bacto tryptose, 2% yeast extract, 0.1% thioglycolate, pH 7.4) broth. The cultures were back diluted to OD_600_ = 0.02 and grown in pre-reduced TY broth supplemented with ethanol or with oleic acid in ethanol. Final concentrations of oleic acid varied as shown in the figure (0, 0.05, 0.15, 0.6 and 3 mM). (**A**) Growth in the presence of 0.6 mM oleic acid or the ethanol vehicle was monitored by taking samples at various times and reading OD_600_. One experiment representative of 3 independent experimental trials is shown. (**B**) Survival after 25 h of culture with the indicated concentration of oleic acid was measured by plating. Each symbol indicates the results from an independent culture and the bar is the geometric mean. (one-way ANOVA with post hoc Dunnett’s multiple comparisons test *, *p* = 0.003; **, *p* < 0.0001). (**C**) *C. difficile* cells grown for 8 or 24 h with the indicated concentration of oleic acid were harvested in RNAprotect. RNA was extracted and the gene transcription was measured in cDNA and normalized to *rpoA* transcription. Each symbol indicates results from an independent culture and the bar is the geometric mean. (one-way ANOVA with post hoc Dunnett’s multiple comparisons test *, *p* < 0.02; **, *p* < 0.0003). (**D**) Samples were treated as in (C). Each symbol indicates the results from an independent culture and the bar is the geometric mean. (*t* test, **, *p* < 0.0003).

Gene transcription and the growth of *C difficile* in pre-reduced TY broth with or without oleic acid was analyzed at different concentrations. The exponential phase growth of *C. difficile* in TY broth containing 0.6 mM oleic acid (0.017% *w/v*) was not markedly altered in comparison to cultures containing the vehicle control (Figure 6A). However, when the cells transitioned to the stationary phase, a decrease in optical density was observed for the cultures grown with oleic acid. The decrease became more pronounced at 24 h of culturing. Plating cultures after 25 h of growth showed that CFU/mL declined in an oleic acid concentration-dependent manner (Figure 6B). Additionally, the spore levels after 51 h of culture were measured and found not to be affected by oleic acid (Supplementary Figure S8). These results demonstrated that while oleic acid did not reduce the growth rate of *C. difficile*, it compromised the viability of *C. difficile* bacterial cells during the stationary phase. 

Furthermore, growth in the media containing oleic acid resulted in the reduced transcription of the toxin genes. *C. difficile* cells were grown in a pre-reduced TY broth supplemented with oleic acid at various concentrations or vehicle control and the bacteria were harvested using RNAprotect. RNA was extracted and the gene transcription was measured in cDNA. Transcription of *tcdB* was reduced in the presence of either 0.05 mM or 0.6 mM oleic acid grown for 8 and 24 h of culture (Figure 6C) in comparison with the vehicle control. Similar effects were observed at 1 and 9 mM oleic acid (Supplementary Figure S9). Transcription of *tcdA* and *tcdR* showed a similar reduction (Figure 6D, 8 h of culture with or without 0.6 mM oleic acid). In contrast, other genes such as *fliC* and *clpB* (CDR20291_1933) were not changed by the presence of oleic acid (Figure 6D, 8 h of culture, 0.6 mM oleic acid). These results support the model in which growth in the presence of oleic acid reduced the toxin gene transcription and stationary phase survival of *C. difficile*. Both of these effects could contribute to reducing the virulence of *C. difficile* in the olive oil-fed mice.

## 4. Discussion

The host metabolic milieu is influenced by a variety of factors, including resident microbiota. Our studies show that the opportunistic pathogenic fungus and common colonizer of the gastrointestinal tract, *C. albicans*, influences the metabolic composition of the host gastrointestinal tract. This study focused on lipid species because compounds in the linoleic and linolenic acid metabolism pathway were enriched among compounds showing at least a trend towards higher levels in *C. albicans*-colonized mice (Figure 1). Previously, we have shown that the pre-colonization of antibiotic-treated mice with *C. albicans* protects against a lethal *C. difficile* challenge [51]. This protection appeared to be conferred by an increase in *IL-17* transcription induced by the presence of *C. albicans*. Furthermore, we were able to recapitulate the protection without *C. albicans* pre-colonization by exogenous IL-17 administration. In our current study, we show that olive oil feeding is able to protect mice from lethal CDI, to a lesser extent than *C. albicans* colonization, without inducing an increase in *IL-17* transcription. Combined, these studies highlight a potential protective role for *C. albicans* during CDI by both modifying the metabolic milieu and inducing host immunological responses, which are antagonistic to *C. difficile*.

Although these studies suggest a protective role for *C. albicans* during CDI, other groups have reported opposite effects. Panpetch and co-workers recently reported that the presence of *C. albicans* during CDI increased virulence in a murine model [67]. In these studies, the authors introduced *C. albicans* one day before *C. difficile* infection and observed disease exacerbation presumably due to increased inflammatory responses [67]. The conflicting results from these murine studies are likely due to the timing of *Candida* acquisition. It has previously been shown that *C. albicans* is able to colonize and shape the host gastrointestinal microbiota [68,69]. Moreover, long-term colonization allows *C. albicans* to become integrated into the host microbiota and shape its microbial, metabolic and immune environment. Overall, the conflicting results from the animal studies and clinical observations described above probably reflect a multitude of factors including the distinct times of acquisition of the two organisms since timing affects disease outcome in animal studies, differences in historic antibiotic usage in each patient, and differences in microbial strains [70]. Additionally, in clinical observation studies, the presence of *Candida* colonization before CDI was not assessed.

In our current study, olive oil feeding reduced the killing of antibiotic-treated mice in response to *C. difficile* spore challenge (Figure 2). As described above, the toxin production by *C. difficile* induces severe damage to the host gastrointestinal tract. Olive oil feeding resulted in lower levels of transcription of the toxin gene *tcdB* in bacteria associated with the cecum epithelium (Figure 3A). Furthermore, the culture of *C. difficile* in the presence of oleic acid, a primary component of olive oil, resulted in the reduced transcription of *tcdB* and reduced the viability of *C. difficile* cells in the stationary phase (Figure 6). These findings support the model in which increased levels of unsaturated fatty acids in the lower gastrointestinal tract reduce the virulence of *C. difficile* cells, through direct effects on gene transcription in bacteria associated with the epithelium, contributing to the ability of mice to survive an otherwise lethal challenge with *C. difficile* spores.

Oleic acid has been shown to affect a variety of microorganisms [71,72]. More recently, Subramanian and co-workers showed that oleic acid inhibits the virulence and biofilm formation of *C. albicans* [73]. In these studies, oleic acid was shown to disrupt *C. albicans* biofilms and significantly inhibit filamentation. Additionally, oleic acid induced oxidative stress responses in *C. albicans* and displayed broad effects, which included the modification of the ergosterol composition on the fungal cell membrane although the exact effects in an animal model are not known. A related unsaturated fatty acid, linoleic acid, has recently been shown to enhance *Salmonella enterica* gastrointestinal colonization dissemination to the spleen in a murine model [74]. Both of these studies suggest that lipids could play a complex role in the host gastrointestinal tract, providing beneficial or detrimental effects to the host, possibly influenced by the overall microbiota composition and state of the host immune response.

Interestingly, the levels of lipid species have been shown to rise during CDI. In a murine model, Fletcher et al. characterized the gut metabolome during active infection [75]. These studies showed large metabolic changes during disease. A variety of lipid species increased in abundance in the cecum 30 h post challenge with *C. difficile*. The lipids identified were derived from the host and their increase in abundance was presumably due to toxin damage caused by TcdA and TcdB. Additionally, the increase in abundance of lipids was accompanied by inflammatory mediators. The results reported here suggest that responses of *C. difficile* to lipid species could influence the course of infection.

Other results have shown that feeding a high-fat diet coincident with antibiotic treatment increases susceptibility to CDI [76,77]. The regimen used in our studies was distinct from those previously described. Olive oil feeding was initiated two weeks after the antibiotic treatment and relatively low levels were used. Mice did not gain significant weight due to the oil feeding (Supplementary Figure S3). Furthermore, high-fat diet feeding typically has a strong effect on gut microbiota composition whereas the olive oil feeding used here had a minimal effect (Figure 4). The differences in the amount of fat given and the timing of the intervention probably account for the difference in the effects of high-fat diets versus oil feeding.

The mechanism for the effects of oleic acid on *tcdB* transcription is unknown but in the soil bacterium *Bacillus subtilis*, DNA binding by the *B. subtilis* regulatory protein BscR is inhibited by oleic acid in a gel shift assay [78], showing that DNA binding activity can be altered by a fatty acid. In addition, *tcdB* and *tcdA* are regulated by the sporulation regulator Spo0A [79,80] and the histidine kinases that activate Spo0A [80,81]. The activity of *B. subtilis* KinA (one of the *B. subtilis* sporulation histidine kinases) is directly inhibited by oleic acid [82]. Therefore, oleic acid could be directly affecting a regulator of *tcdB* and *tcdA*. These studies from our group [51] and others [70,83,84] highlight a significant role for the mycobiome during CDI, an area that is deserving of further exploration. 

## Supplementary Materials

The following are available online at https://www.mdpi.com/2309-608X/6/3/100/s1, Figure S1: Quality control of the designed primers. Figure S2: Pre-amplification increases the sensitivity of detection. Figure S3: *C. albicans* colonization. Figure S4: Relative weight of the mice after receiving olive oil for 6 days. Figure S5: Relative weights of the mice on the days following challenge with *C. difficile* spores. Figure S6: Histology of the *C. difficile*-infected murine tissues. Figure S7: Cecal microbiota community diversity scores. Figure S8: Vegetative cells and spores after growth in a TY broth for 51 h with and without oleic acid. Figure S9: Transcription of *tcdB* normalized to *rpoA*. Table S1: Primers used. Table S2: Compounds present at higher or lower levels in cecum contents from the *C. albicans*-colonized mice versus the uncolonized mice. Table S3: Survival of the mice after feeding oils, pilot experiment.
