# Supplementary material for: Lipid Species in the GI Tract are Increased by the Commensal Fungus Candida albicans and Decrease the Virulence of Clostridioides difficile
Source: J Fungi (Basel). 2020 Jul 3;6(3):100. doi: 10.3390/jof6030100 (PMC7557729; doi:10.3390/jof6030100)
Supplement: Supplementary file 1 [file jof-06-00100-s001.zip › Supplementary_Materials/Table S3_oils survival.docx]

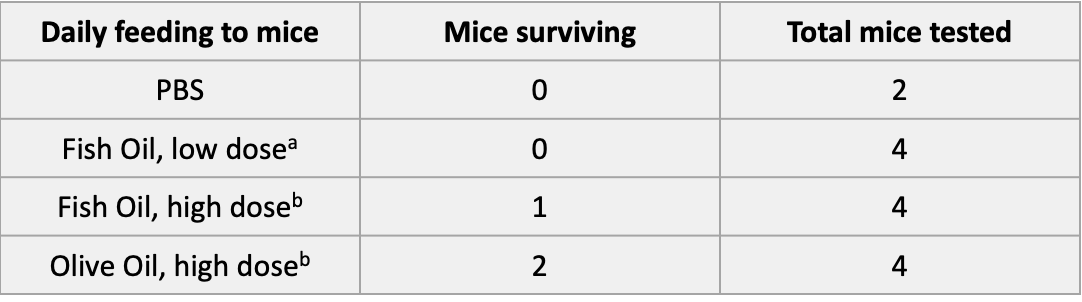


^a^Low dose, 0.5 µl of oil, given orally once per day for 7 days before *C. difficile* challenge and continued until the end of the experiment.

^b^High dose, 3.3 µl of oil, given orally once per day for 7 days before *C. difficile* challenge and continued until the end of the experiment.

**Table S1**. Survival of mice after feeding oils, pilot experiment
