# Supplementary material for: Lipid Species in the GI Tract are Increased by the Commensal Fungus Candida albicans and Decrease the Virulence of Clostridioides difficile
Source: J Fungi (Basel). 2020 Jul 3;6(3):100. doi: 10.3390/jof6030100 (PMC7557729; doi:10.3390/jof6030100)
Supplement: Supplementary file 1 [file jof-06-00100-s001.zip › Supplementary_Materials/Romo_Supplemental_Figures_Legends ckCLEAN.docx]

**Figure S1.** Quality control of designed primers.

Circles, Results with serial 10-fold dilutions of a standard. Each concentration run in triplicate and Ct for the triplicate determinations are plotted. Triangles, two uninfected cecum samples, run in triplicate. All uninfected samples except one sample gave no signal detected after 45 cycles. One uninfected sample for *clpB* gave signal with Ct = 42.

**Figure S2.** Pre-amplification increases sensitivity of detection.

cDNA samples from *C. difficile* cells grown in laboratory culture were diluted to produce a sample with weak signal. This sample was then serially diluted in 4-fold steps and half of each diluted sample was pre-amplified with one PCR primer for 12 cycles. The remaining half was unamplified and diluted to the same concentration as the pre-amplified half. The samples were then analyzed in triplicate by standard qRT PCR for 45 cycles. The Ct for each of the triplicates is plotted. Open triangles indicate samples where no signal was detected. The R^2^ for each sample set is shown, indicating that the pre-amplified samples maintained their quantitative relationship and the sensitivity of detection was improved. One experiment representative of 3 experimental trials is shown.

**Figure S3.** *C. albicans* colonization. *C. albicans* CFU/gm in fecal pellets collected from mice 17 days

post-inoculation with *C. albicans.* Fresh fecal pellets were collected from each mouse, homogenized in PBS and plated on YPD-SA agar medium to emumerate C. albicans CFU*.* Each symbol shows results from an individual mouse and the bar indicates the geometric mean. Open symbol indicates colonization below the limit of detection.

**Figure S4.** Relative weight of mice after receiving olive oil for 6 days.

Mice were treated as in Figure 2A. On the first day of olive oil or PBS feeding, mice were weighed prior to feeding. Mice were weighed every other day during feeding and their relative weights after 6 days of feeding is shown. Each symbol indicates an individual mouse and the bar indicates the average. Circles, antibiotic-treated, uncolonized; triangles, antibiotic-treated, *C. albicans-*colonized.

**Figure S5**. Relative weights of mice on days following challenge with *C. difficile* spores.

Each symbol shows an individual mouse with its weight relative to its weight on the day of spore inoculation (day 0). Bar indicates the average for surviving mice on that day.

**Figure S6**. Histology of *C. difficile-*infected murine tissues.

Mice were treated as described in Figure 2A. On day 2 post-inoculation with spores, mice were sacrificed and their ceca were removed. The cecum wall was fixed in buffered formalin. Processing and H & E staining were conducted by the Tufts Comparative Pathology Core facility and samples were photographed with the 10x objective. (A) Cecum of a PBS-fed mouse, showing an area of edema with infiltration (indicated with bracket). (B) Cecum of an olive oil-fed mouse, showing an area of edema with infiltration (bracket). (C) Histopathological scores of tissues from treatment groups. Scoring was conducted blinded on 30-40 10x fields of view for each tissue. Scores were acquired by dividing the number of fields showing infiltration/inflammation by the total number of fields for that tissue. Each point on the graph represents an individual mouse. A non-parametric t-test was used to compare the groups.

**Figure S7**. Cecal microbiota community diversity scores.

Mice were treated as described in Figure 2A. On day 2 post-inoculation with spores, mice were sacrificed and their ceca were removed. Bacterial composition was analyzed as described in Materials and Methods with QIIME 2 and diversity scores were calculated. Bar represents the mean diversity score for all mice within a group. (A) Chao1, (B) Shannon, and (C) Simpson. Circles, antibiotic-treated, uncolonized; triangles, antibiotic-treated, *C. albicans-*colonized.

**Figure S8.** Vegetative cells and spores after growth in TY broth for 51 hours with and without oleic acid.

Cultures were grown in an anaerobic chamber for 51 hours. Vegetative cells (Veg) were enumerated by plating on BHIS plates. Spores were enumerated by heating the culture to 60^o^C for 10 min. and plating on BHIS plates with taurocholate. Each symbol indicates results from a different culture and the bar indicates the geometric mean.

**Figure S9.** Transcription of *tcdB* normalized to *rpoA*.

*C. difficile* cells were cultured in TY broth with the indicated concentration of oleic acid for 24 hrs in an anaerobic chamber. Cells were harvested and RNA was extracted. Transcription of genes was measured in cDNA using real time RT PCR. Each symbol indicates results from a different culture and the bar indicates the geometric mean. **, p<0.0001 (ANOVA, Dunnett’s multiple comparisons test).
