# Supplementary material for: Lipid Species in the GI Tract are Increased by the Commensal Fungus Candida albicans and Decrease the Virulence of Clostridioides difficile
Source: J Fungi (Basel). 2020 Jul 3;6(3):100. doi: 10.3390/jof6030100 (PMC7557729; doi:10.3390/jof6030100)
Supplement: Supplementary file 1 [file jof-06-00100-s001.zip › Supplementary_Materials/Table S2_metabolites.docx]

**Table S2.** Compounds present at higher or lower levels in cecum contents from *C. albicans*-colonized mice vs. uncolonized mice.

| Compound | Fold change (*C. albicans* colonized/uncolonized)^a^ | p value *(t* test)^a^ |
| --- | --- | --- |
| D-urobilin | 52.786 | 0.022373 |
| I-urobilinogen | 32.642 | 0.051301 |
| 3-methyladipate | 6.4917 | 0.030451 |
| 2-aminophenol sulfate | 5.1816 | 0.042638 |
| propionylcarnitine (C3) | 4.3797 | 0.020525 |
| vanillactate | 4.3414 | 0.0022462 |
| urea | 4.2962 | 0.049213 |
| guanosine | 3.9329 | 0.015767 |
| kynurenate | 3.724 | 0.01976 |
| 2-myristoylglycerol (14:0) | 3.7054 | 0.063508 |
| 2,3-dihydroxyisovalerate | 3.654 | 0.054343 |
| p-cresol sulfate | 3.5613 | 0.058866 |
| N1-methyladenosine | 3.4196 | 0.011082 |
| syringic acid | 3.0344 | 0.015 |
| 1-palmitoyl-GPS (16:0) | 2.9628 | 0.063046 |
| N-acetylneuraminate | 2.9406 | 0.04744 |
| N-glycolylneuraminate | 2.9036 | 0.049654 |
| xanthosine | 2.8803 | 0.037997 |
| inosine | 2.8422 | 0.037678 |
| 12-dehydrocholate | 2.771 | 0.0057527 |
| 5-aminovalerate | 2.7317 | 0.039319 |
| N-acetylleucine | 2.6951 | 0.067519 |
| N1-methylinosine | 2.6892 | 0.051527 |
| prolylglycine | 2.5888 | 0.072694 |
| docosapentaenoate (n3 DPA; 22:5n3) | 2.5722 | 0.078558 |
| N-acetylisoleucine | 2.5319 | 0.093137 |
| sinapate | 2.4829 | 0.03093 |
| erythrose | 2.2154 | 0.045699 |
| 10-heptadecenoate (17:1n7) | 2.2144 | 0.086355 |
| indolelactate | 2.154 | 0.068891 |
| N-acetylglucosaminylasparagine | 2.1124 | 0.028233 |
| N1-methylguanosine | 2.0911 | 0.017333 |
| N-acetylmethionine | 2.0785 | 0.028358 |
| glutarate (C5-DC) | 2.016 | 0.056289 |
| malate | 1.9814 | 0.053322 |
| myristate (14:0) | 1.98 | 0.069562 |
| dodecanedioate (C12-DC) | 1.9757 | 0.034171 |
| 2-isopropylmalate | 1.9538 | 0.098255 |
| 3-hydroxyphenylacetate | 1.8922 | 0.09767 |
| vanillate | 1.8554 | 0.079979 |
| N-formylmethionine | 1.7824 | 0.033142 |
| dimethylarginine (SDMA + ADMA) | 1.7421 | 0.0060736 |
| indoleacetate | 1.7143 | 0.079373 |
| docosahexaenoate (DHA; 22:6n3) | 1.7081 | 0.034003 |
| beta-alanine | 1.703 | 0.049029 |
| 1-palmitoyl-GPE (16:0) | 1.6899 | 0.039423 |
| N6-carbamoylthreonyladenosine | 1.6863 | 0.077814 |
| phenylpyruvate | 1.646 | 0.042918 |
| stearoyl sphingomyelin (d18:1/18:0) | 1.6328 | 0.016725 |
| glycylleucine | 1.6321 | 0.075757 |
| 1-margaroylglycerol (17:0) | 1.6179 | 0.087581 |
| thiamin (Vitamin B1) | 1.6164 | 0.022822 |
| 1-linoleoyl-GPC (18:2) | 1.5849 | 0.034091 |
| 3-methyl-2-oxovalerate | 1.5822 | 0.090831 |
| 2'-O-methyluridine | 1.5808 | 0.042446 |
| 1,5-anhydroglucitol (1,5-AG) | 1.5778 | 0.016555 |
| eicosenoate (20:1) | 1.5723 | 0.091806 |
| eicosapentaenoate (EPA; 20:5n3) | 1.5666 | 0.081395 |
| succinate | 1.5598 | 0.082767 |
| sulfate | 1.5369 | 0.026289 |
| pyridoxate | 1.5236 | 0.0776 |
| 2-oxindole-3-acetate | 1.4532 | 0.041134 |
| 1-stearoylglycerol (18:0) | 1.444 | 0.09695 |
| N-acetylhistamine | 1.4379 | 0.096866 |
| linolenate [alpha or gamma; (18:3n3 or 6)] | 1.4187 | 0.052322 |
| stachydrine | 1.3922 | 0.062425 |
| creatinine | 1.375 | 0.022658 |
| 1-palmitoyl-GPC (16:0) | 1.34 | 0.06442 |
| N1-Methyl-4-pyridone-3-carboxamide | 1.3379 | 0.0701 |
| hexadecanedioate (C16-DC) | 1.2722 | 0.011222 |
| 1-methyl-4-imidazoleacetate | 1.2538 | 0.049654 |
| linoleate (18:2n6) | 1.2501 | 0.073797 |
| ergosterol | 1.2476 | 0.034721 |
| glutamate | 1.2445 | 0.096298 |
| stigmasterol | 1.2377 | 0.082791 |
| gamma-aminobutyrate (GABA) | 1.2218 | 0.062279 |
| 3-hydroxymyristate | 1.2091 | 0.034846 |
| Compound | Fold change (*C. albicans* colonized/uncolonized)^a^ | p value *(t* test)^a^ |
| dimethylglycine | 0.0018036 | 0.033212 |
| coprostanol | 0.023704 | 0.0042436 |
| 3-(3-hydroxyphenyl)propionate | 0.20551 | 0.07389 |
| N2-acetyllysine | 0.22851 | 0.019784 |
| 2-hydroxypalmitate | 0.24767 | 0.011412 |
| adenine | 0.25272 | 0.052696 |
| O-sulfo-L-tyrosine | 0.2699 | 0.073212 |
| 1-methylguanine | 0.31489 | 0.003734 |
| 2-hydroxynervonate | 0.34088 | 0.00077516 |
| N2,N6-diacetyllysine | 0.35369 | 0.052221 |
| xanthine | 0.37715 | 1.27E-05 |
| N-acetylglutamine | 0.39422 | 0.029068 |
| inositol hexakisphosphate | 0.39943 | 0.052365 |
| 2-hydroxybehenate | 0.40573 | 0.00067327 |
| sarcosine | 0.41448 | 0.074733 |
| 2-hydroxystearate | 0.42685 | 0.012469 |
| 2-hydroxyarachidate | 0.43741 | 0.002365 |
| ribose | 0.44018 | 0.034566 |
| pipecolate | 0.44174 | 0.049237 |
| 2-aminoadipate | 0.45882 | 0.027482 |
| guanidinoacetate | 0.47307 | 0.019026 |
| phenylalanylalanine | 0.47558 | 0.040111 |
| N-acetylarginine | 0.48914 | 0.056735 |
| tyrosylglycine | 0.49385 | 0.04464 |
| 2-hydroxylignocerate | 0.5164 | 0.0038431 |
| galactitol (dulcitol) | 0.523 | 0.040674 |
| galactonate | 0.54486 | 0.051112 |
| protoporphyrin IX | 0.55275 | 0.05347 |
| tryptophylglycine | 0.56234 | 0.019153 |
| N-formylanthranilic acid | 0.56414 | 0.012119 |
| 1-methyladenine | 0.57157 | 0.0045931 |
| leucylalanine | 0.60712 | 0.021427 |
| phenylalanylglycine | 0.61509 | 0.078621 |
| retinol (Vitamin A) | 0.65076 | 0.062492 |
| hypoxanthine | 0.65626 | 0.074997 |
| 2-oxoadipate | 0.72002 | 0.023186 |
| tartronate (hydroxymalonate) | 0.75112 | 0.026811 |
| quinolinate | 0.75166 | 0.041578 |
| glutarylcarnitine (C5-DC) | 0.76403 | 0.070092 |
| biopterin | 0.77592 | 0.0499 |

^a^Analyzed using Metaboanalyst
