# Supplementary material for: Lipid Species in the GI Tract are Increased by the Commensal Fungus Candida albicans and Decrease the Virulence of Clostridioides difficile
Source: J Fungi (Basel). 2020 Jul 3;6(3):100. doi: 10.3390/jof6030100 (PMC7557729; doi:10.3390/jof6030100)
Supplement: Supplementary file 1 [file jof-06-00100-s001.zip › Supplementary_Materials/Table S1_Primers used.docx]

**Table S1** Primers used

|  | A. *C. difficile* genes |  |  |
| --- | --- | --- | --- |
| Gene | Forward primer | Reverse primer | Source or reference |
| *tcdB* | TTAAATACTATTACAGATGCAGCCAAAG | AATCCTTCAGATAATGTAGGAAGTAAAT | This study |
| *tcdA* | ATATTTATAGTCAGGAGTTGTTAAATCG | TGTTTTAAATAAATCAGAGTGAATACCT | This study |
| *tcdR* | GTCTGTTTTTGAGGAAGATATTTGTTTT | TATTTTTAGCCTTATTAACAGCTTGTCT | This study |
| *rpoA* | GAAAATAAAACAGAGAATGTTCCAATAG | TAAGTTTAAATGCTCAACTAATACCTTT | This study |
| *fliC* | ATGAGTTGTTACAATTAAAGGATGAAGT | TTTAAGCTTGCTATTGTAGAACTTGTTA | This study |
| *clpB* | TAAATATTTTATCACAAGTGAGAGGTAG | GGTCTAATTTATTTTTCTTGGCTAAATC | This study |
| *mcsA* | AAAATATACATGGGCATATAGAACATAC | GCTCTATCTAAATCTTCTTTTAGTTCTT | This study |
|  |  |  |  |
| Gene | *C. difficile* Preamplification primer |  | Source or reference |
| *rpoA* | TTTAGCAATAGCTACATTAGATGATAA |  | This study |
| *fliC* | GGTAATATACTACAAAGAATGAGAACTT |  | This study |
| *mcsA* | AACATATAGTGAGTTTAAGAATAATGGG |  | This study |
|  |  |  |  |
|  | B. Mouse genes |  |  |
| Gene | Forward primer | Reverse primer | Source or reference |
| *Gapdh* | AGGTCGGTGTGAACGGATTTG | TGTAGACCATGTAGTTGAGGTCA | Dror et al, 2007 |
| *Il17a* | GCTCCAGAAGGCCCTCAGA | AGCTTTCCCTCCGCATTGA | Overbergh et al, 2003 |
| *Tnfα* | CATCTTCTCAAAATTCGAGTGACAA | TGGGAGTAGACAAGGTACAACCC | Overbergh et al, 2003 |
| *Il23* | TGTGCCTAGGAGTAGCAGTCCTGA | TTGGCGGATCCTTTGCAAGCAGAA | Paranavitana et al, 2005 |
|  |  |  |  |
|  | C. Eubacteria |  |  |
| rDNA | ACTCCTACGGGAGGCAGCAGT | ATTACCGCGGCTGCTGGC | Barman et al, 2008 |

Barman M, Unold D, Shifley K, Amir E, Hung K, Bos N, Salzman N. 2008. Enteric Salmonellosis Disrupts the Microbial Ecology of the Murine Gastrointestinal Tract. Infect Immun **76**:907-15.

Dror N, Alter-Koltunoff M, Azriel A, Amariglio N, Jacob-Hirsch J, Zeligson S, Morgenstern A, Tamura T, Hauser H, Rechavi G, Ozato K, Levi B-Z. 2007. Identification of IRF-8 and IRF-1 target genes in activated macrophages. Mol Immunol **44**:338-46.

Overbergh L, Giulietti A, Valckx D, Decallonne B, Bouillon R, Mathieu C. 2003. The Use of Real-Time Reverse Transcriptase PCR for the Quantification of Cytokine Gene Expression. J Biomol Tech **14**:33-43.

Paranavitana C, Zelazowska E, Izadjoo M, Hoover D. 2005. Interferon-γ associated cytokines and chemokines produced by spleen cells from *Brucella*-immune mice. Cytokine **30**:86-92.
