# Supplementary figures and images for: Lipid Species in the GI Tract are Increased by the Commensal Fungus Candida albicans and Decrease the Virulence of Clostridioides difficile
Source: J Fungi (Basel). 2020 Jul 3;6(3):100. doi: 10.3390/jof6030100 (PMC7557729; doi:10.3390/jof6030100)

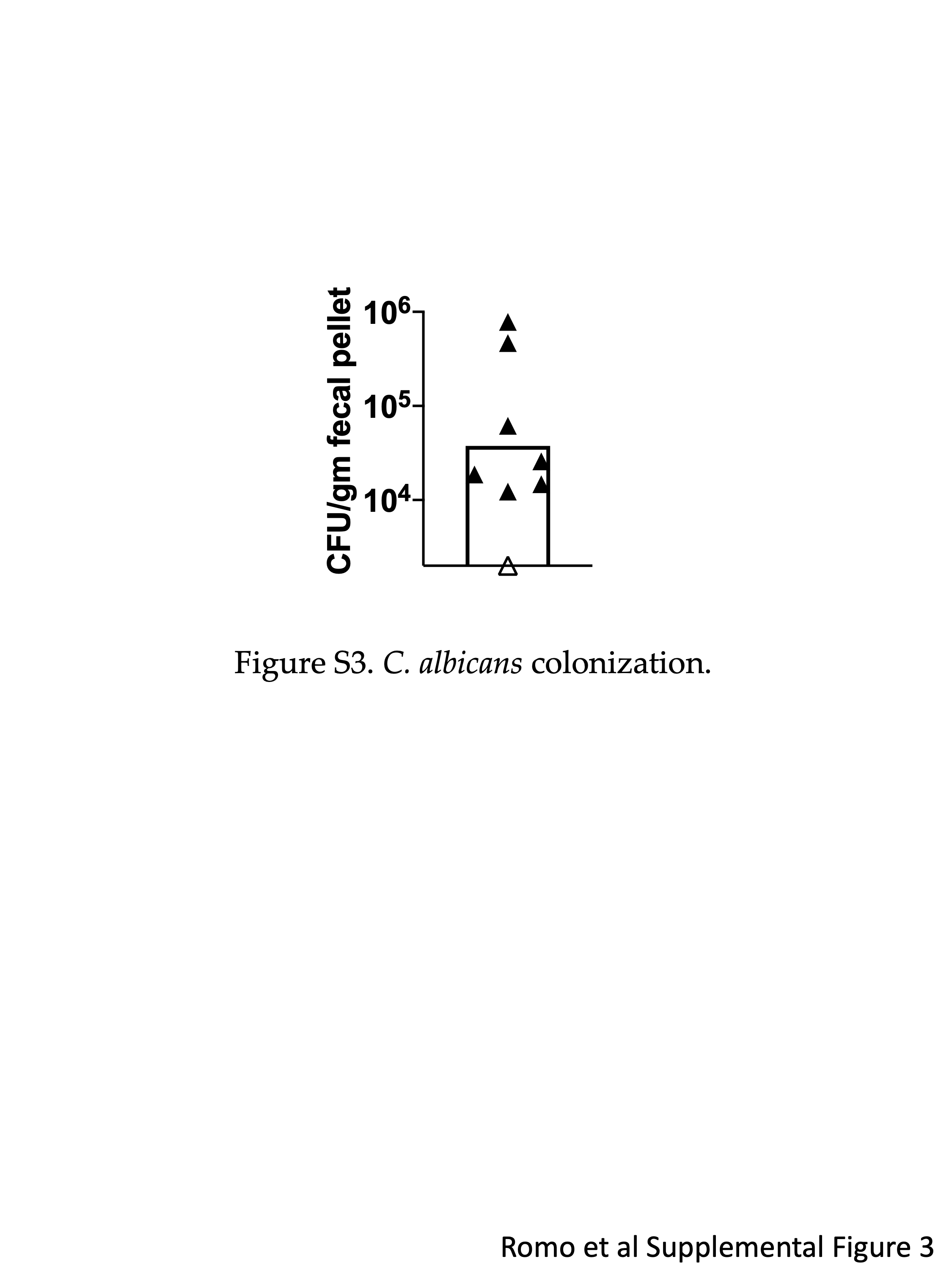

Supplement: Supplementary file 1 [file jof-06-00100-s001.zip › Supplementary_Materials/Supplemental_Figures_TIFF/Fig S3.tiff]

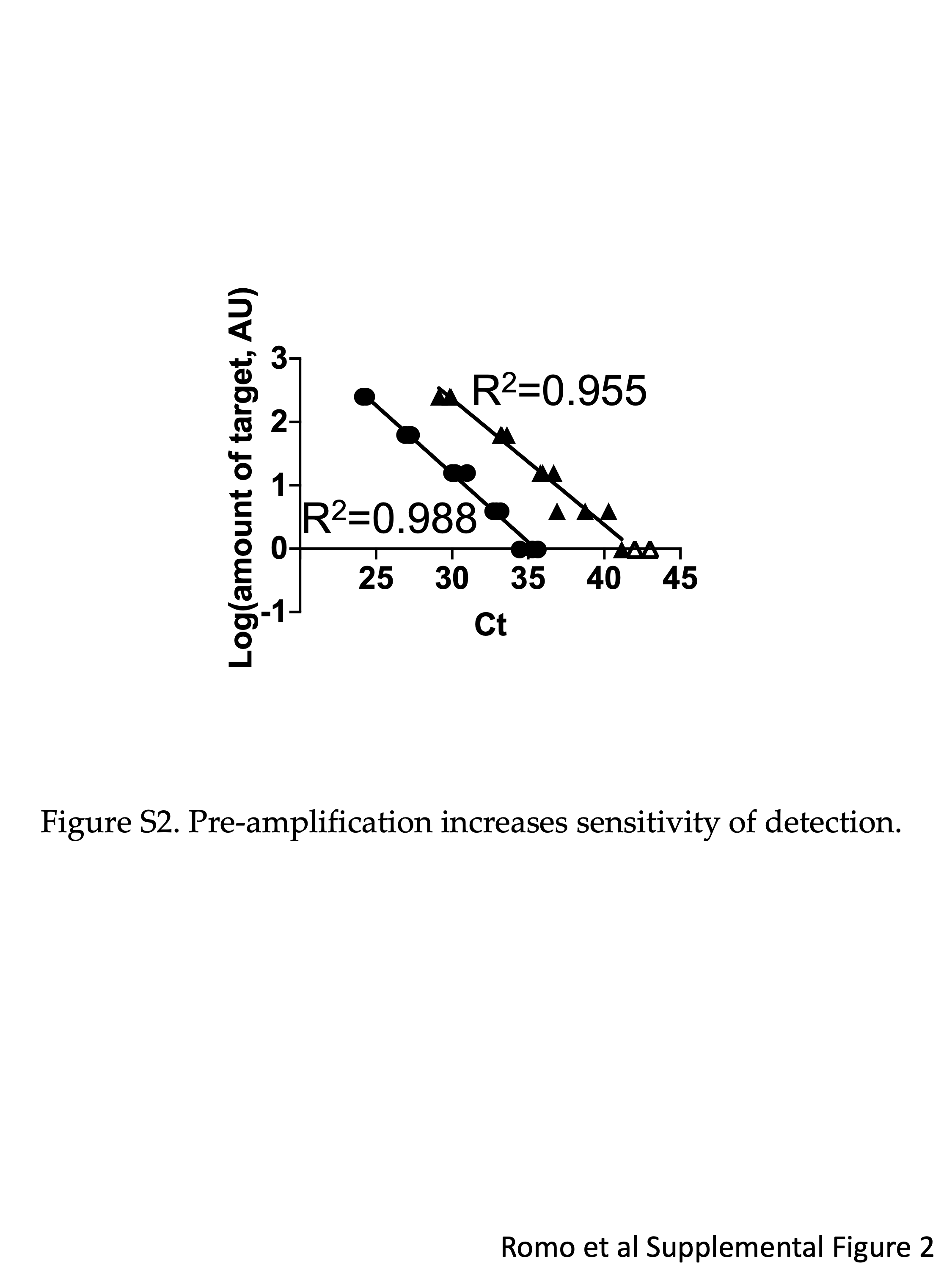

Supplement: Supplementary file 1 [file jof-06-00100-s001.zip › Supplementary_Materials/Supplemental_Figures_TIFF/Fig S2.tiff]

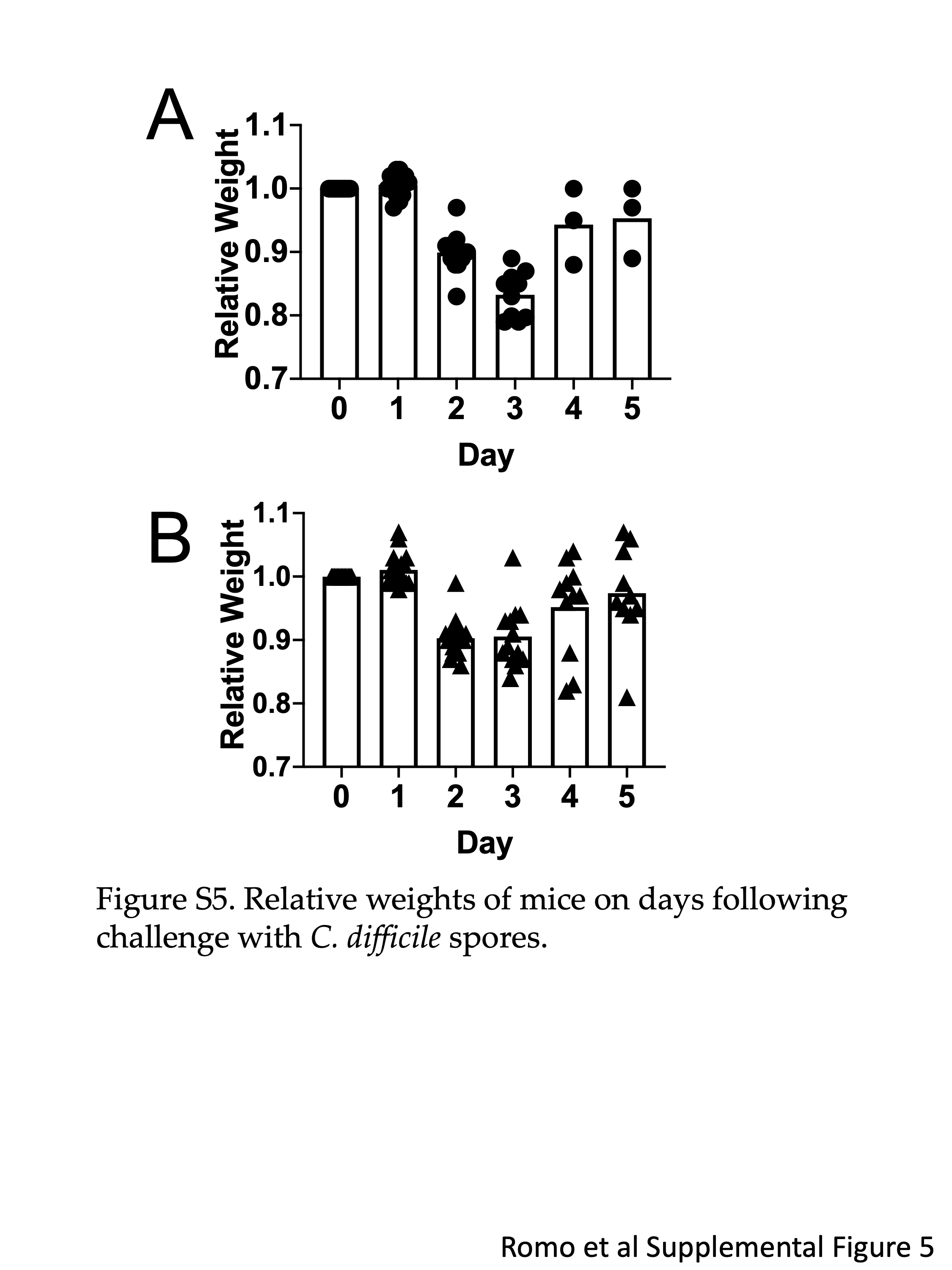

Supplement: Supplementary file 1 [file jof-06-00100-s001.zip › Supplementary_Materials/Supplemental_Figures_TIFF/Fig S5.tiff]

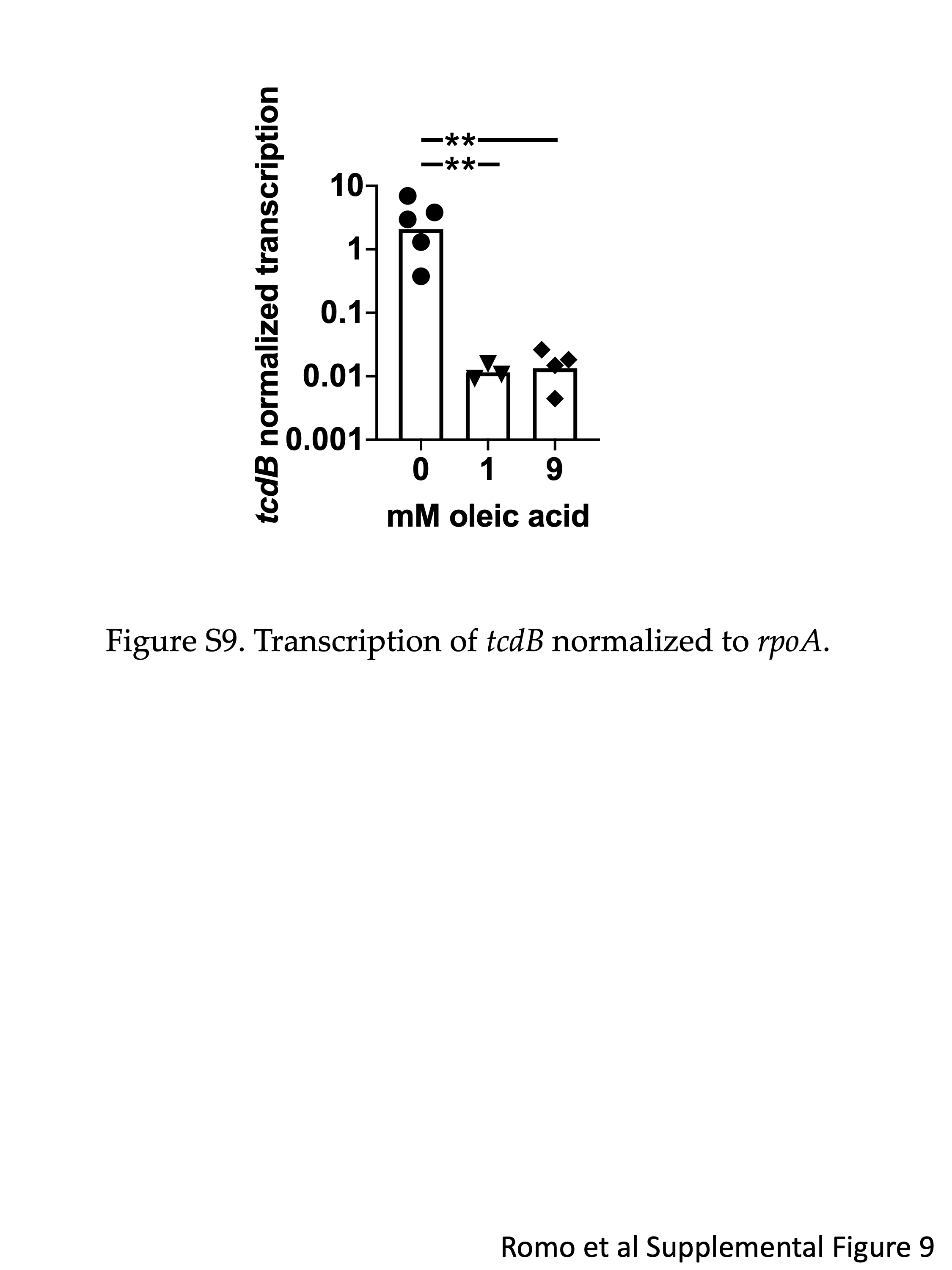

Supplement: Supplementary file 1 [file jof-06-00100-s001.zip › Supplementary_Materials/Supplemental_Figures_TIFF/Fig S9.tiff]

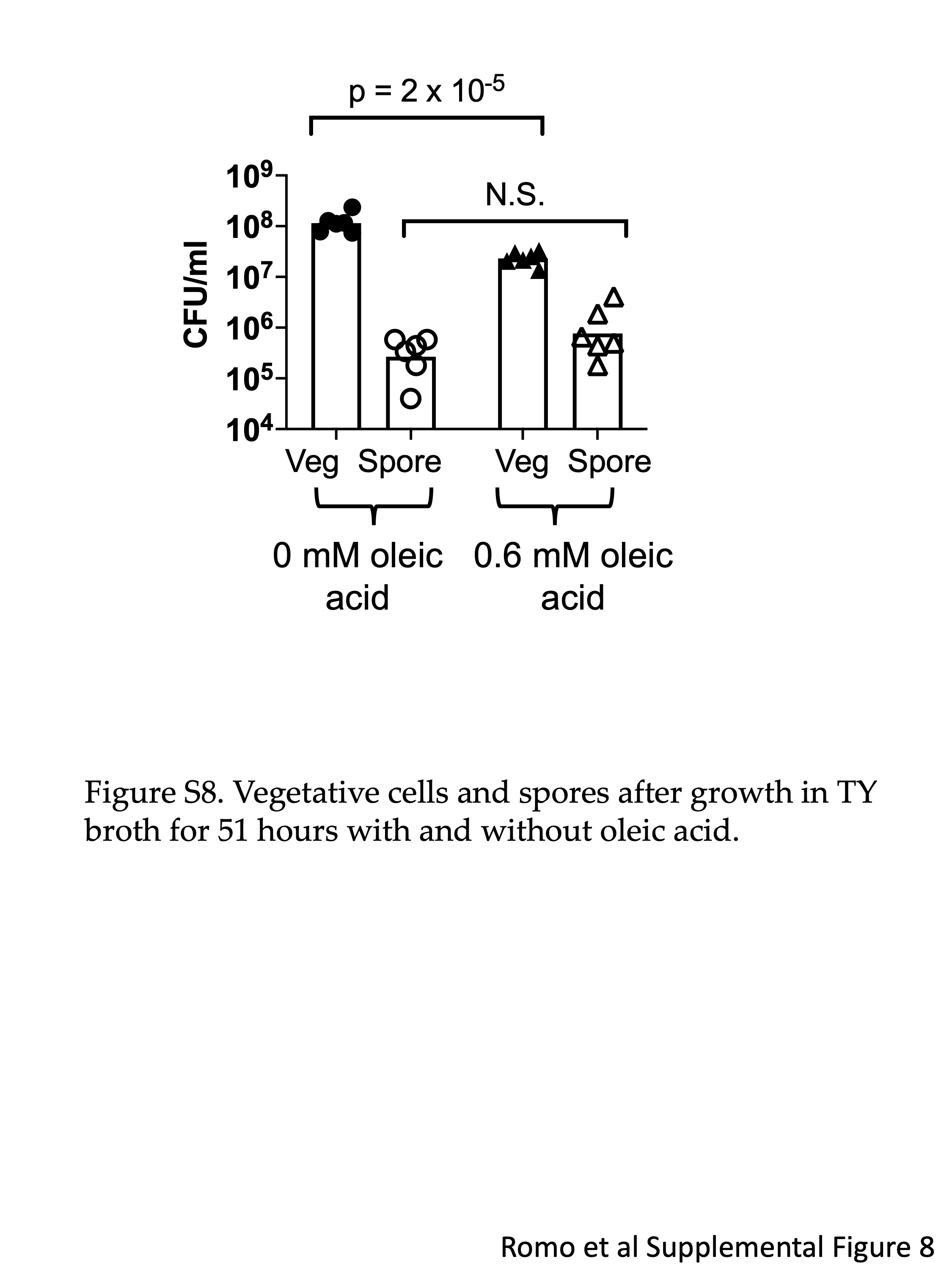

Supplement: Supplementary file 1 [file jof-06-00100-s001.zip › Supplementary_Materials/Supplemental_Figures_TIFF/Fig S8.tiff]

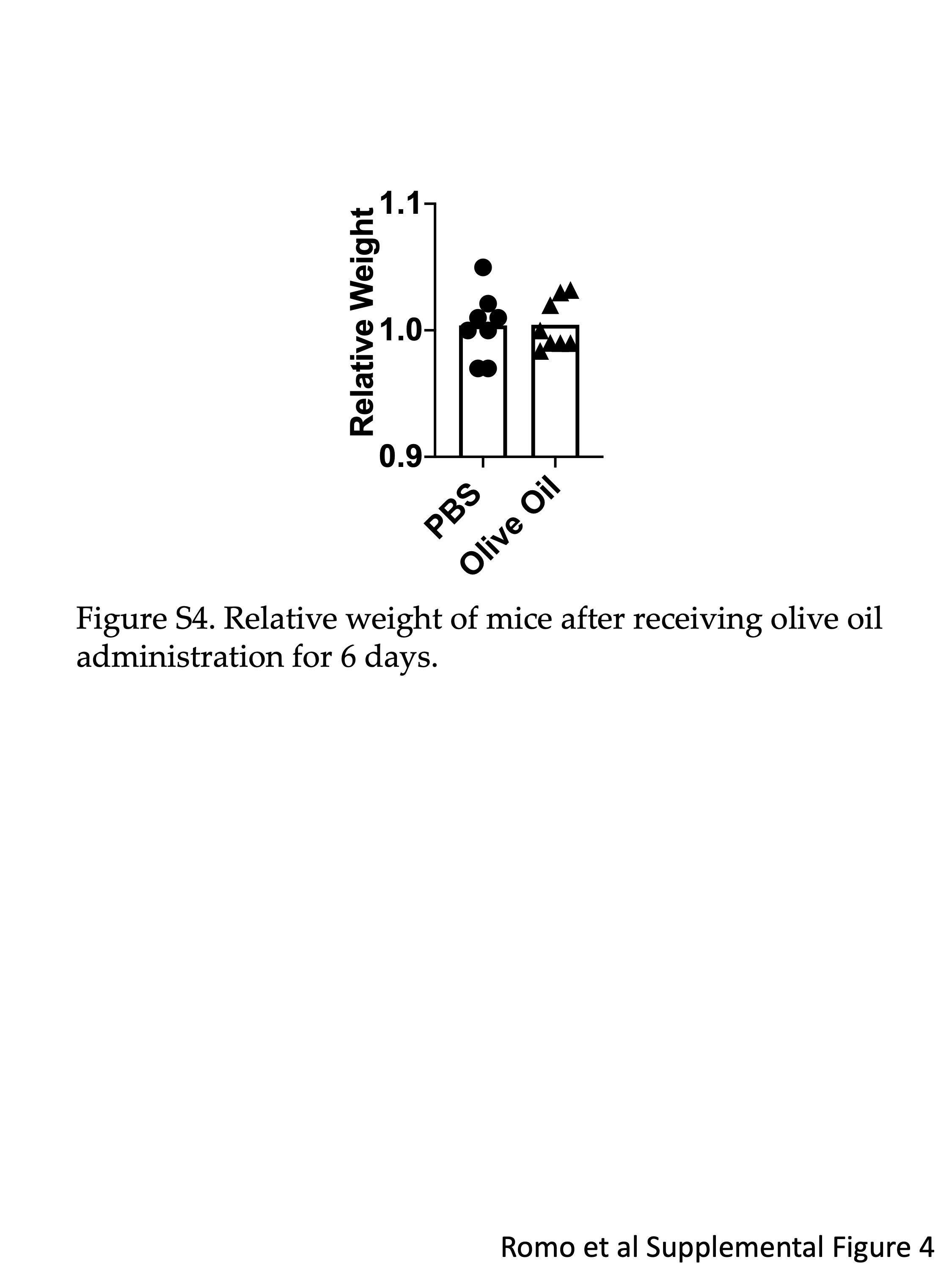

Supplement: Supplementary file 1 [file jof-06-00100-s001.zip › Supplementary_Materials/Supplemental_Figures_TIFF/Fig S4.tiff]

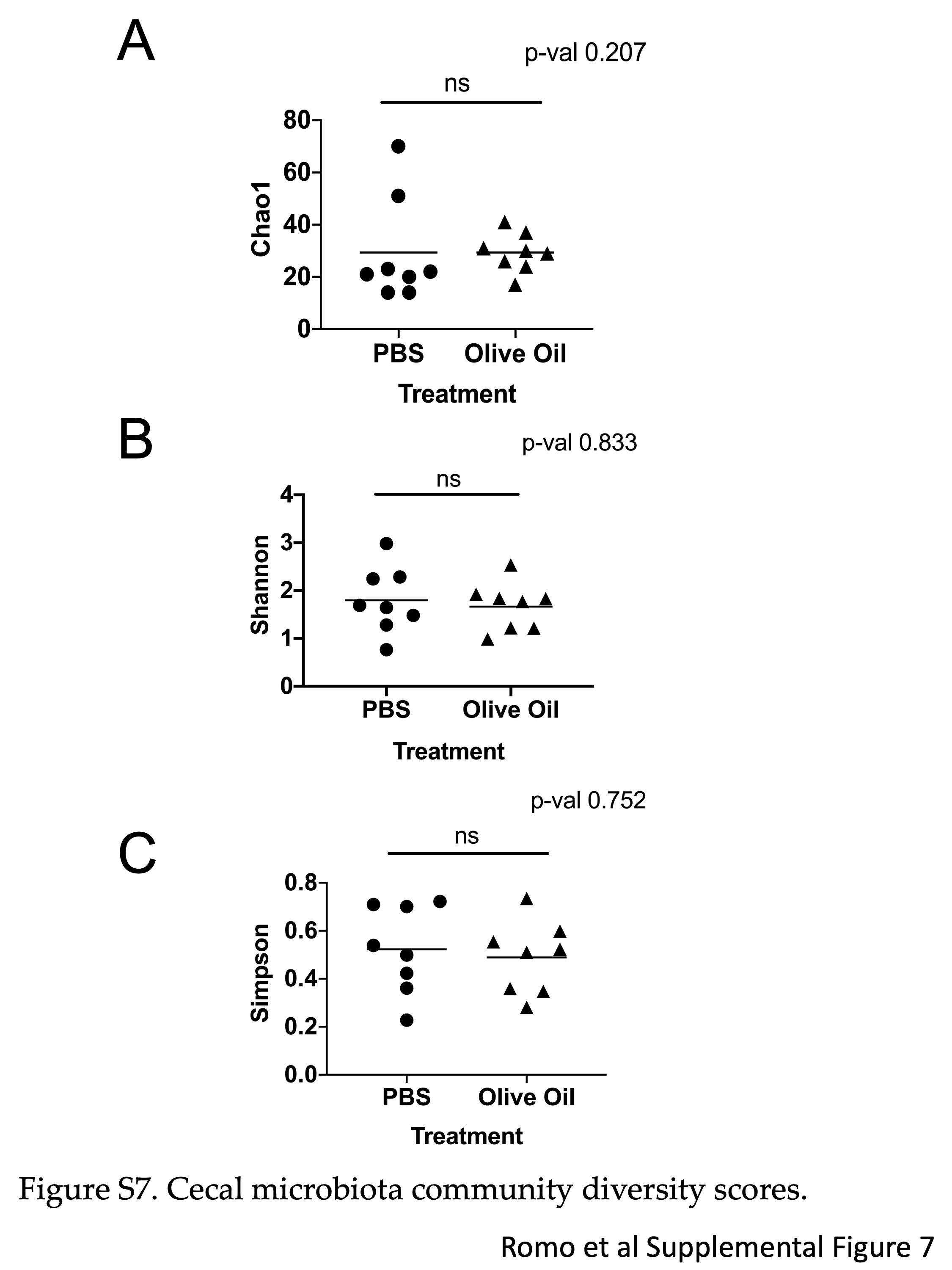

Supplement: Supplementary file 1 [file jof-06-00100-s001.zip › Supplementary_Materials/Supplemental_Figures_TIFF/Fig S7.tiff]

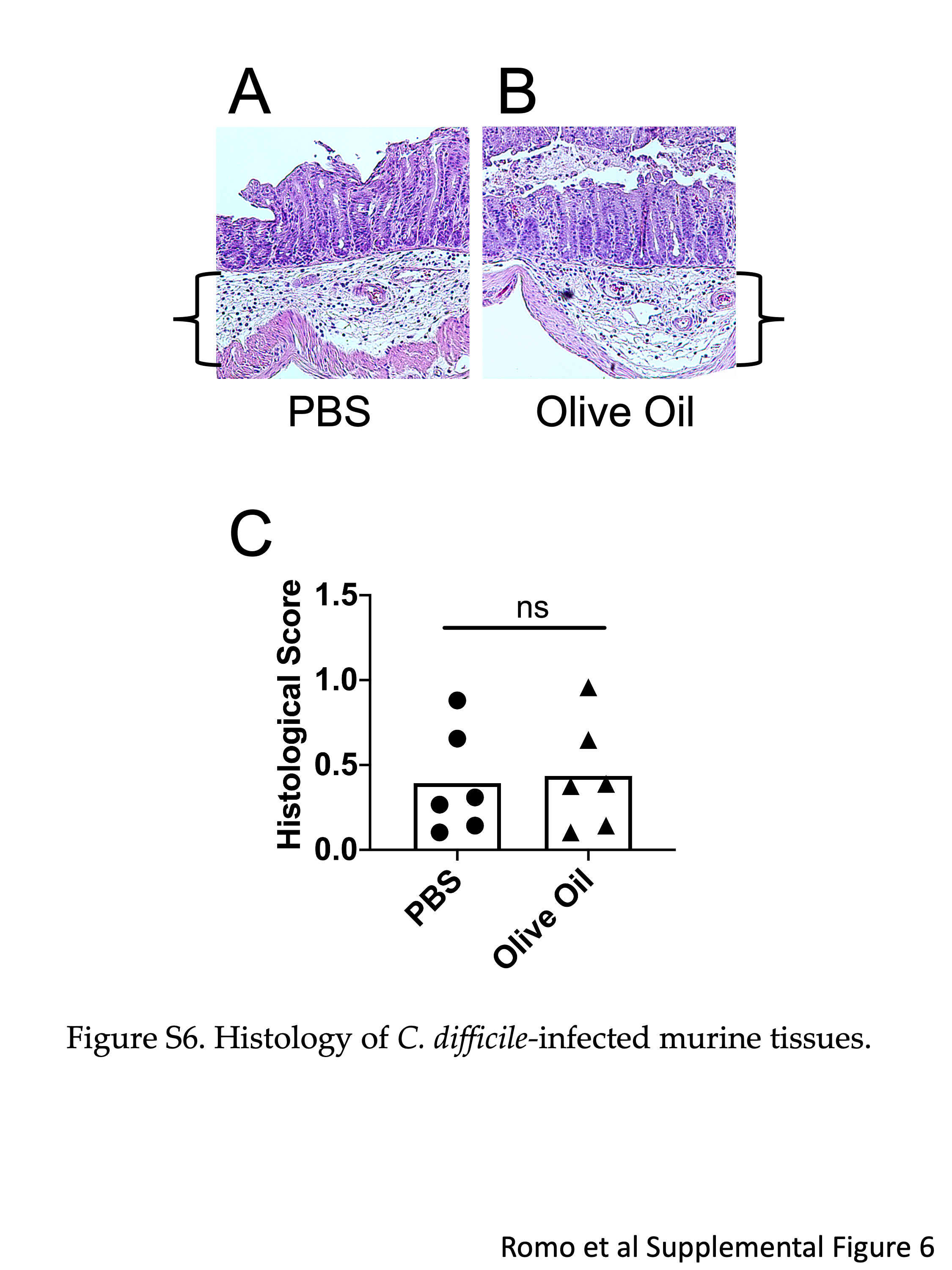

Supplement: Supplementary file 1 [file jof-06-00100-s001.zip › Supplementary_Materials/Supplemental_Figures_TIFF/Fig S6.tiff]

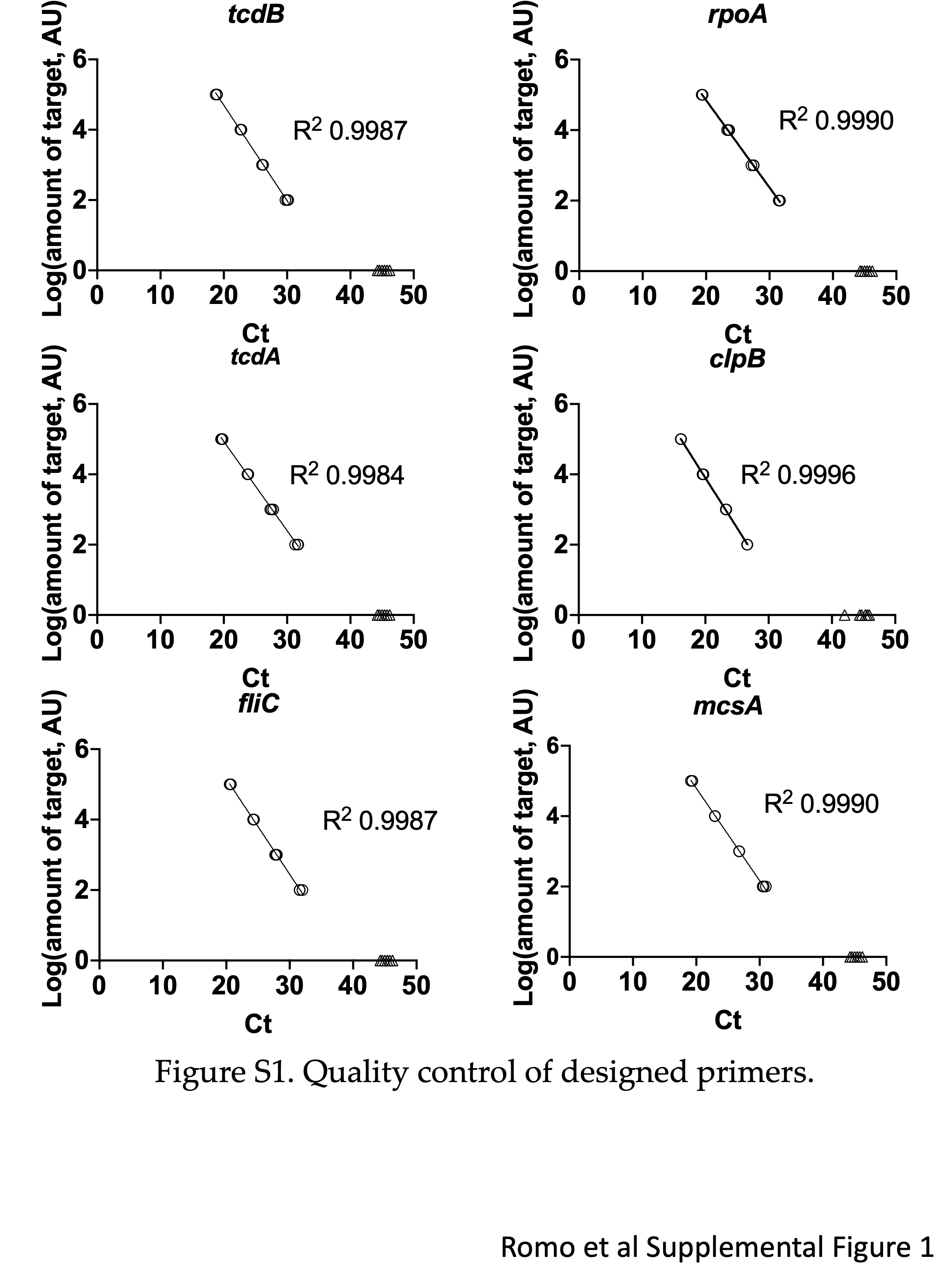

Supplement: Supplementary file 1 [file jof-06-00100-s001.zip › Supplementary_Materials/Supplemental_Figures_TIFF/Fig S1.tiff]

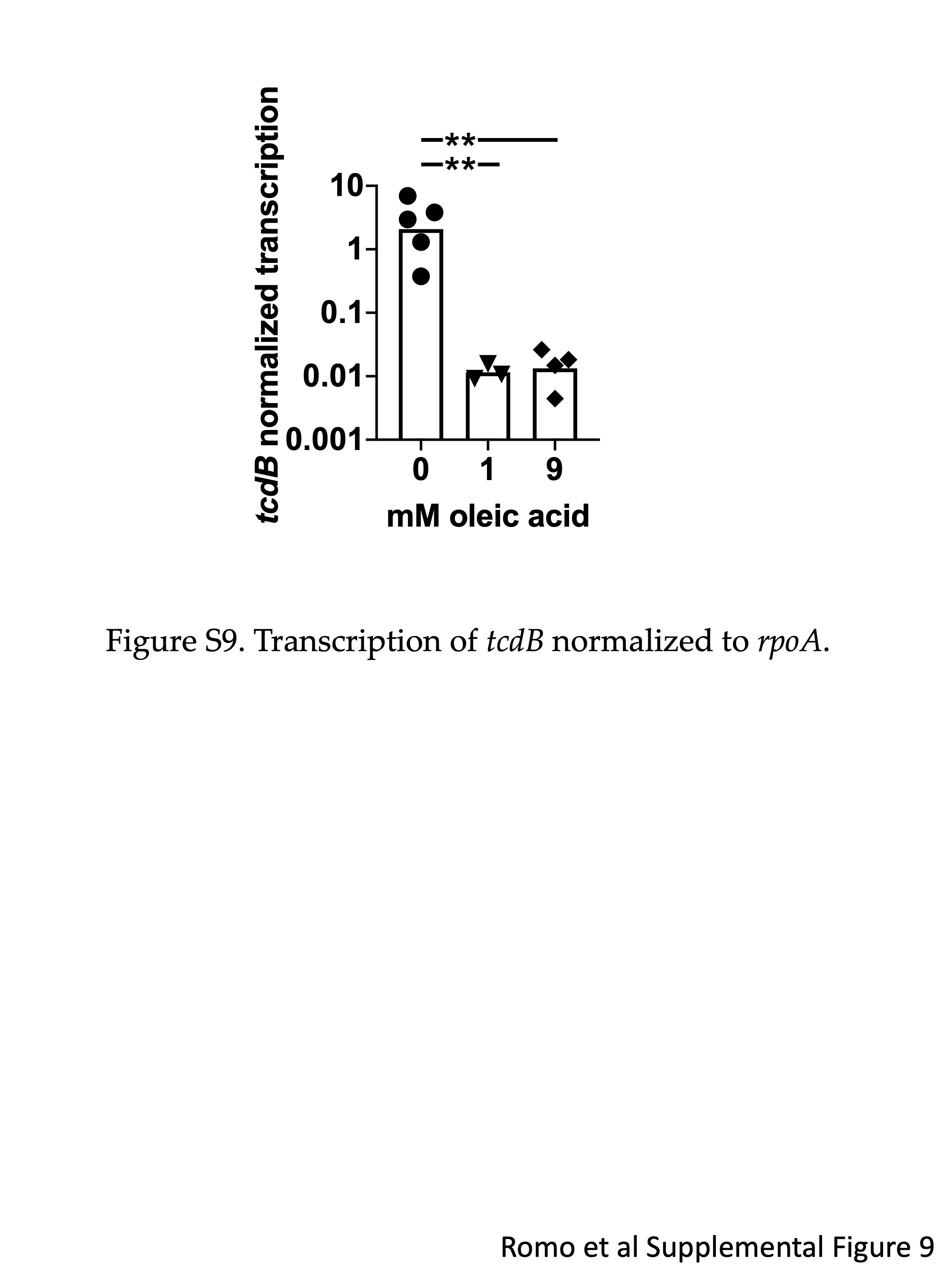

Supplement: Supplementary file 1 [file jof-06-00100-s001.zip › Supplementary_Materials/Supplemental_Figures_JPEG/Fig-S9.jpg]

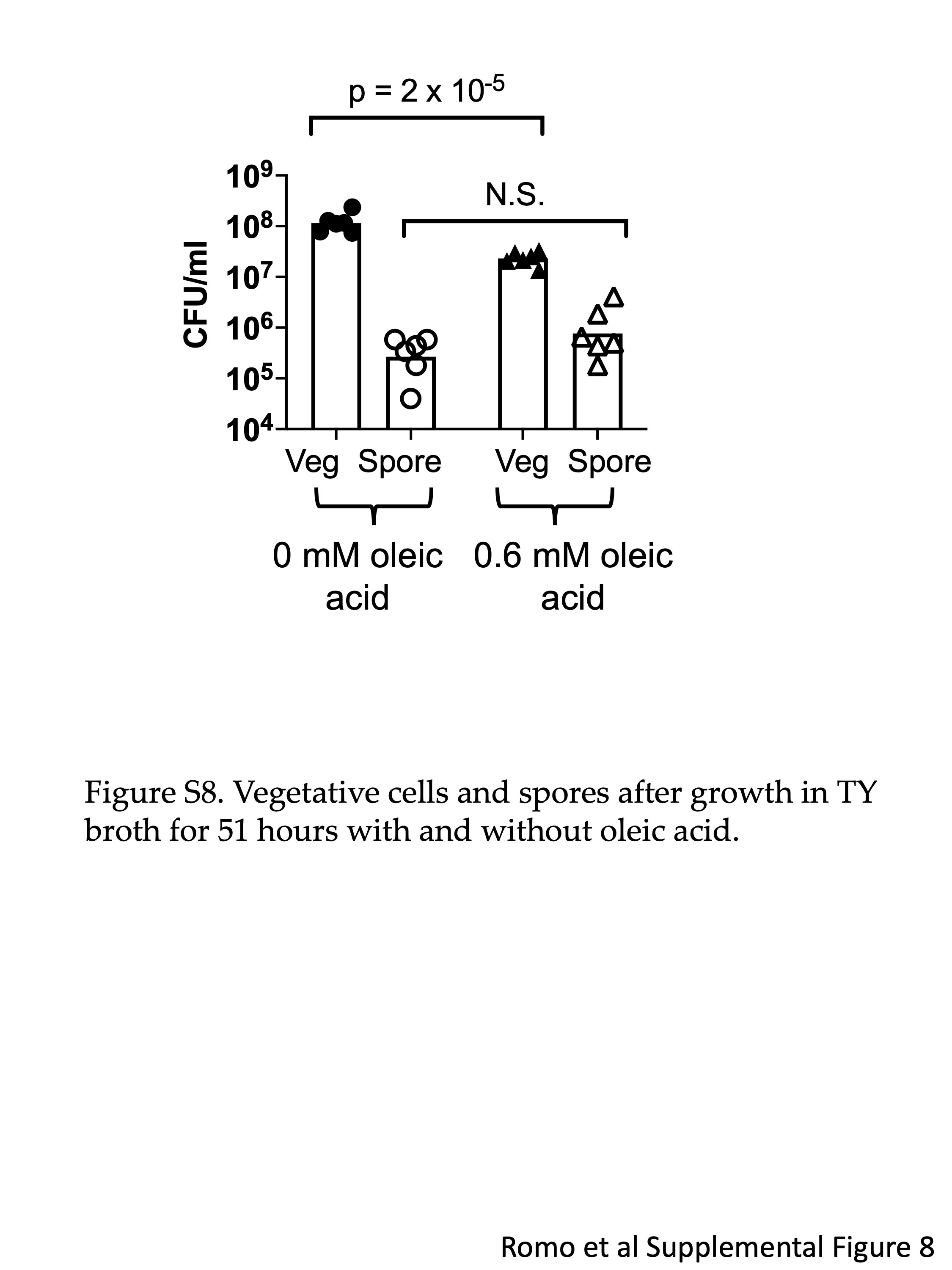

Supplement: Supplementary file 1 [file jof-06-00100-s001.zip › Supplementary_Materials/Supplemental_Figures_JPEG/Fig-S8.jpg]

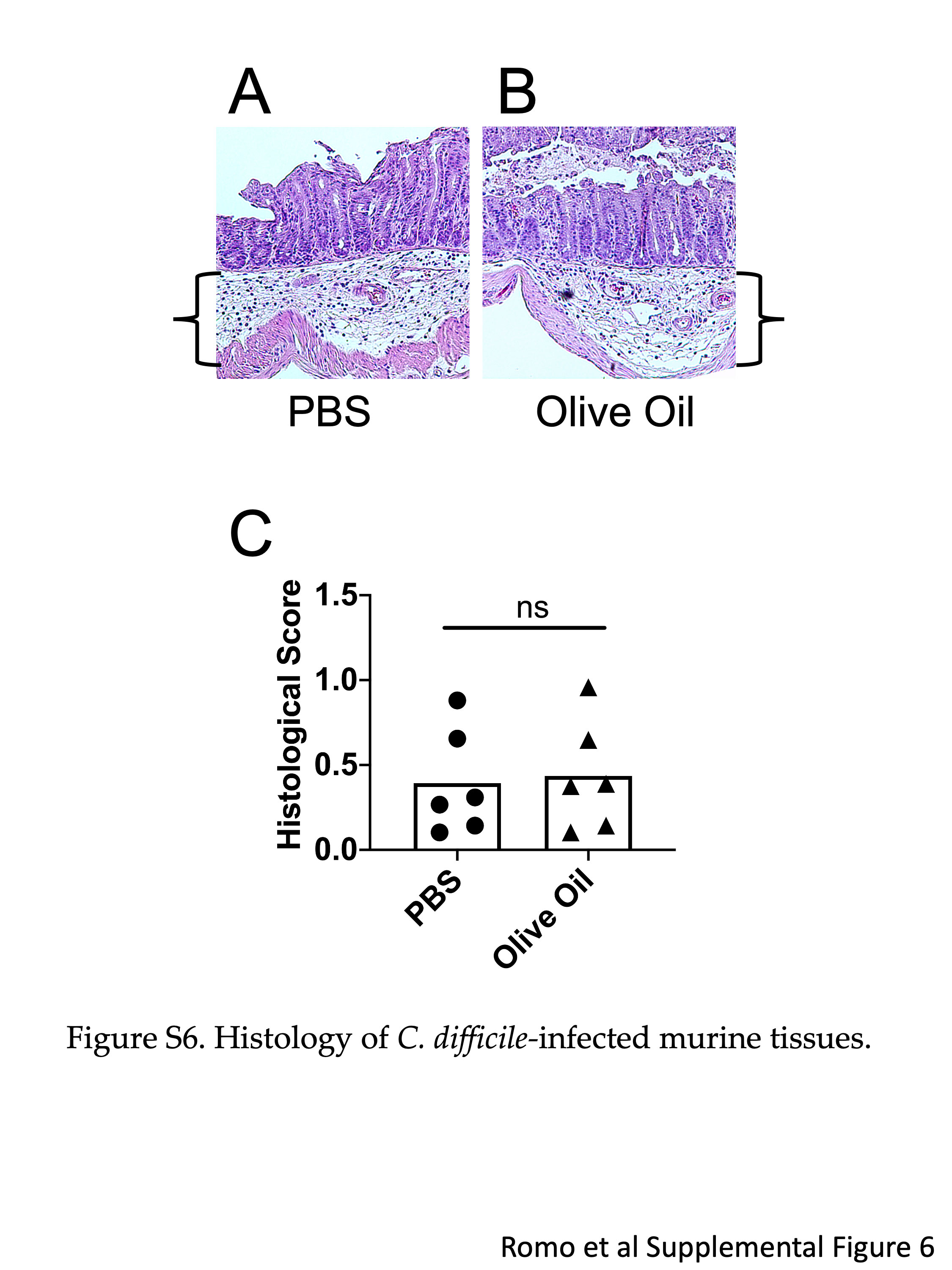

Supplement: Supplementary file 1 [file jof-06-00100-s001.zip › Supplementary_Materials/Supplemental_Figures_JPEG/Fig-S6.jpg]

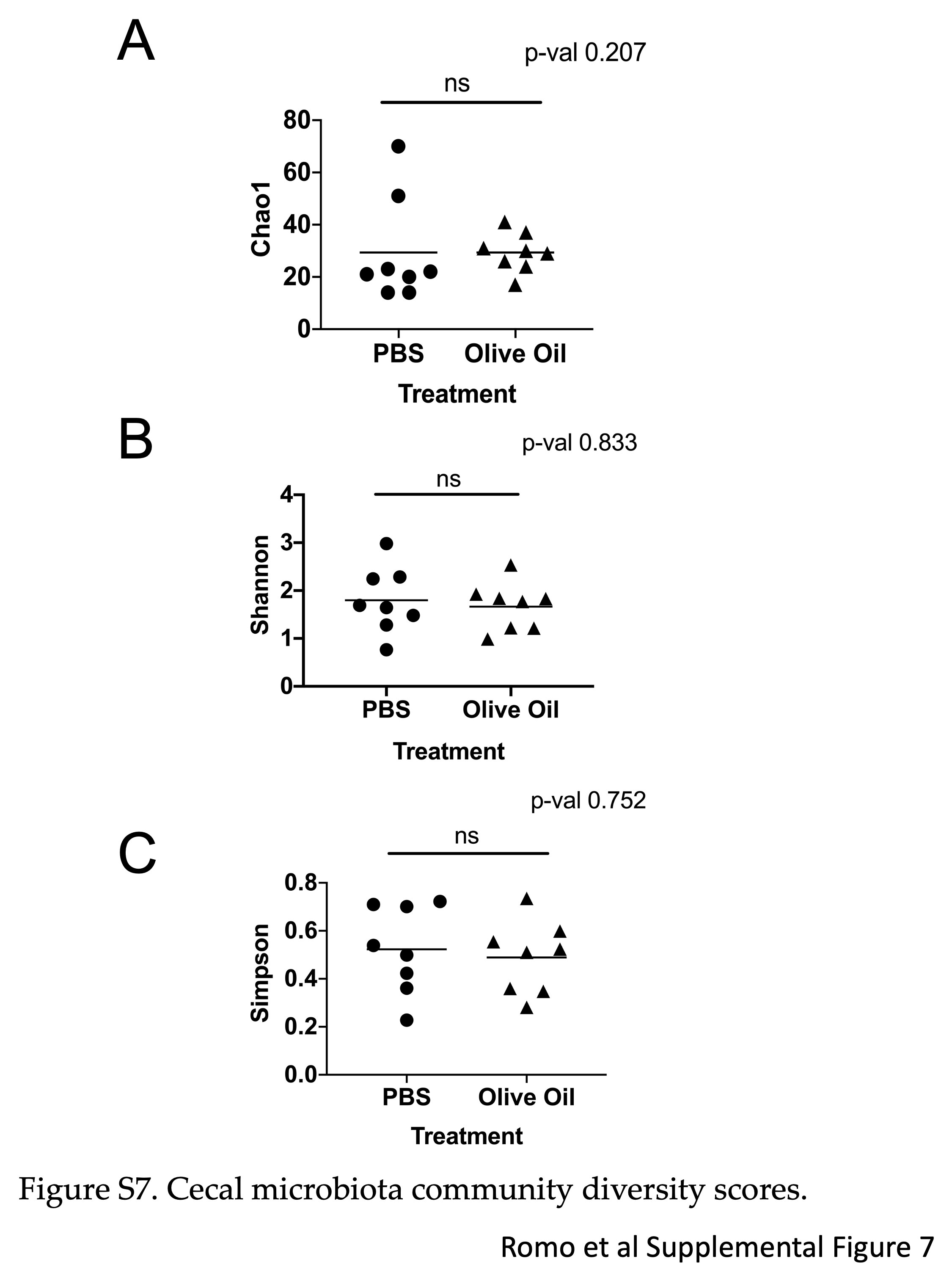

Supplement: Supplementary file 1 [file jof-06-00100-s001.zip › Supplementary_Materials/Supplemental_Figures_JPEG/Fig-S7.jpg]

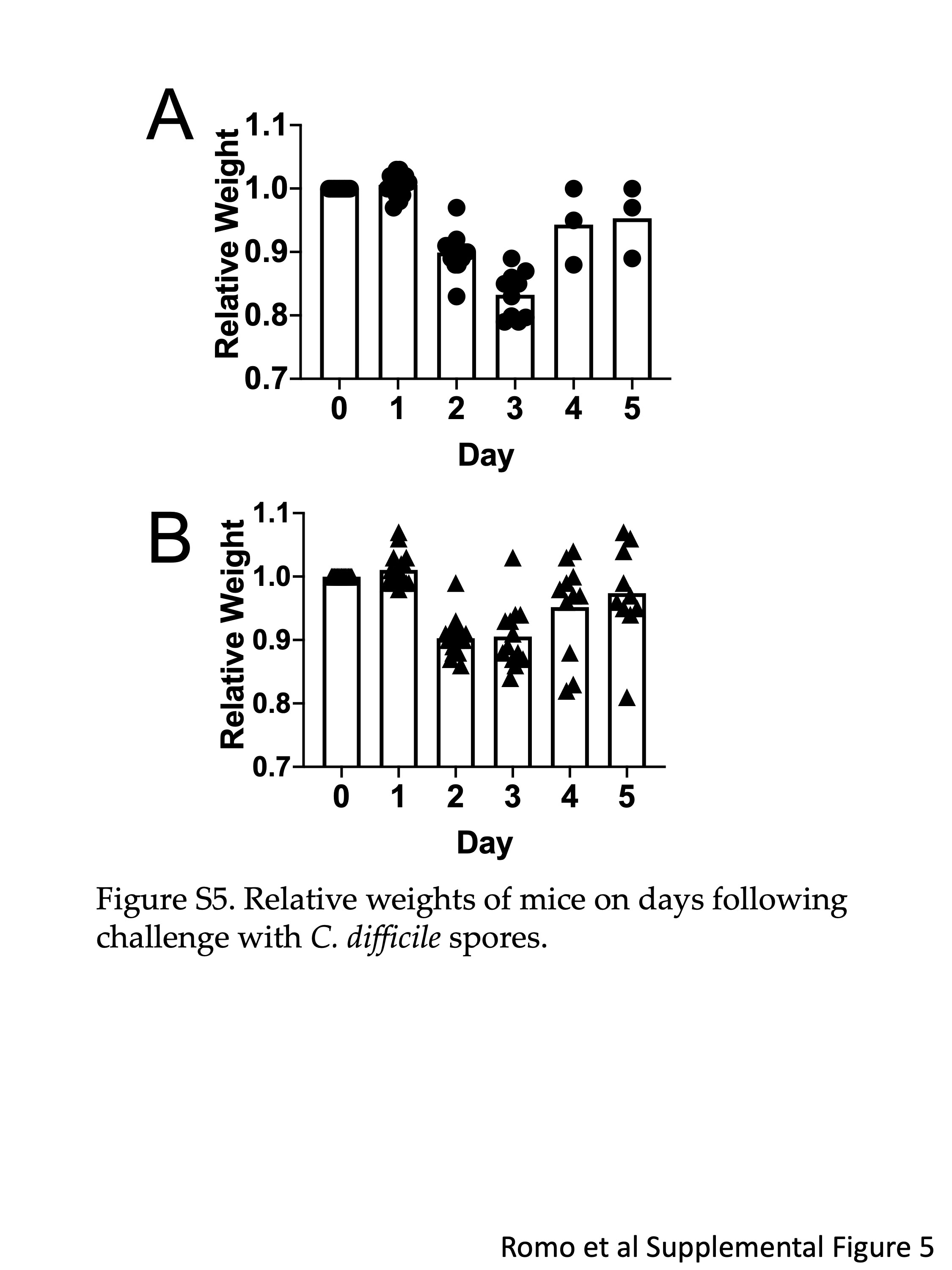

Supplement: Supplementary file 1 [file jof-06-00100-s001.zip › Supplementary_Materials/Supplemental_Figures_JPEG/Fig-S5.jpg]

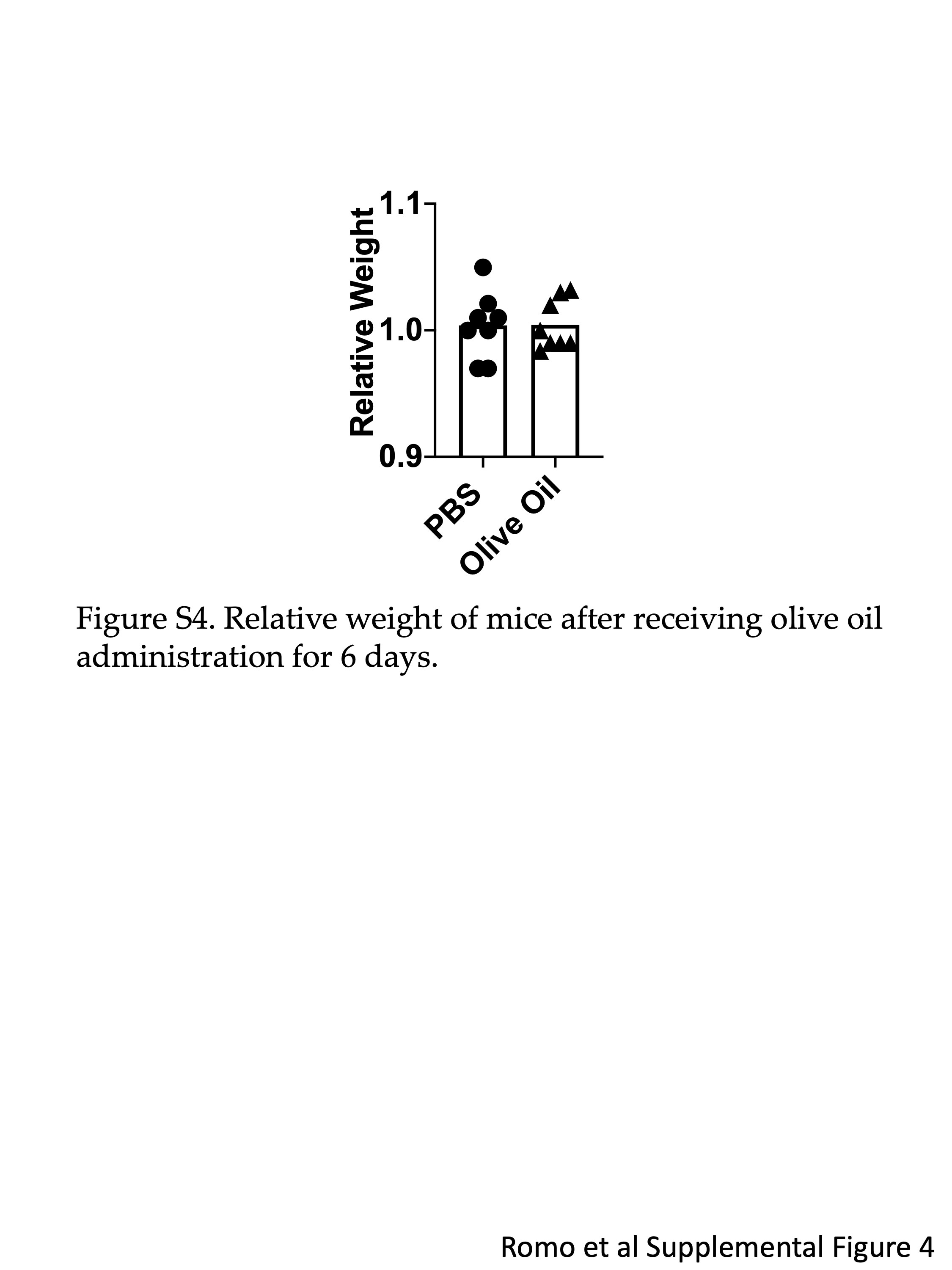

Supplement: Supplementary file 1 [file jof-06-00100-s001.zip › Supplementary_Materials/Supplemental_Figures_JPEG/Fig-S4.jpg]

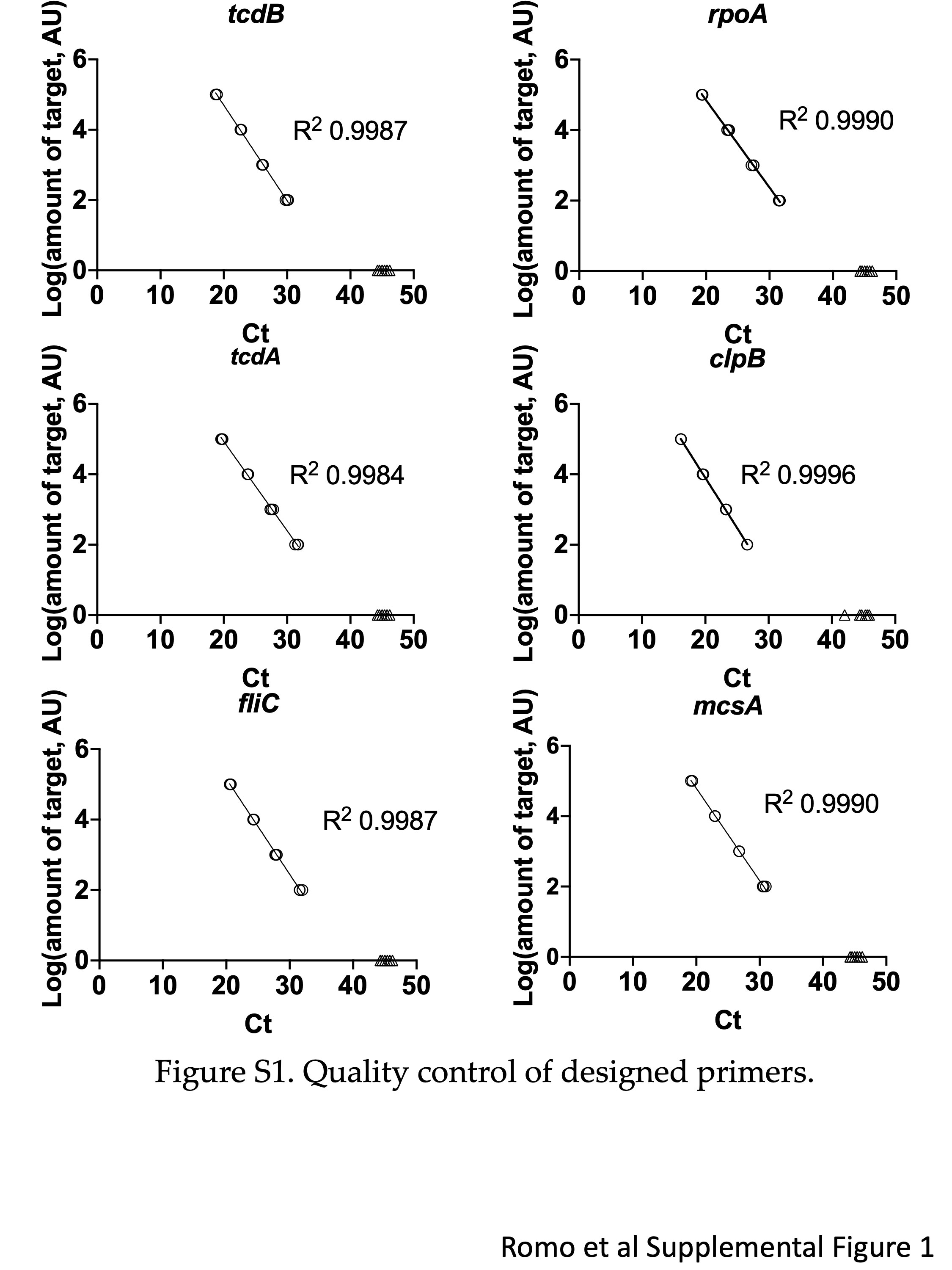

Supplement: Supplementary file 1 [file jof-06-00100-s001.zip › Supplementary_Materials/Supplemental_Figures_JPEG/Fig-S1.jpg]

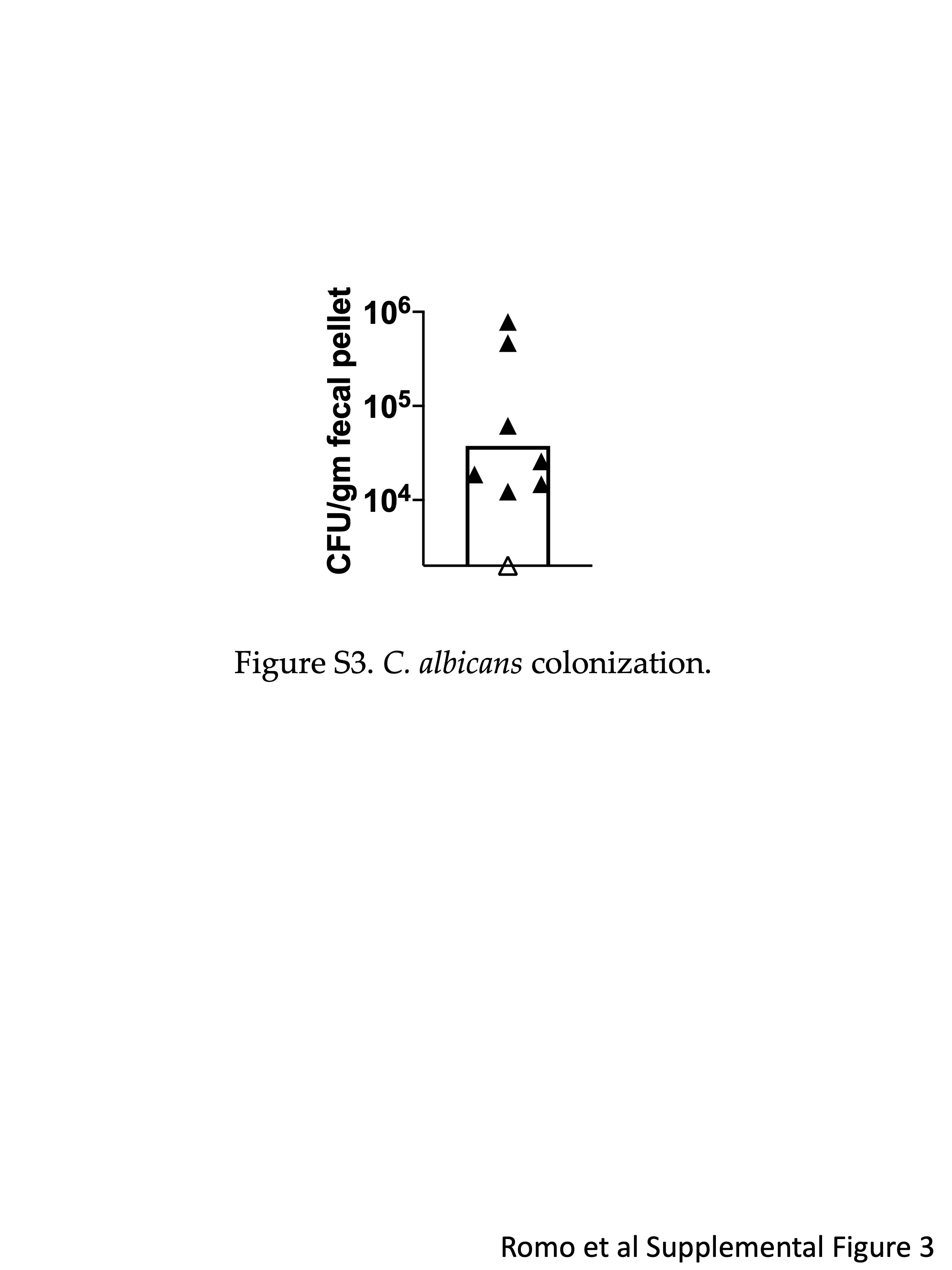

Supplement: Supplementary file 1 [file jof-06-00100-s001.zip › Supplementary_Materials/Supplemental_Figures_JPEG/Fig-S3.jpg]

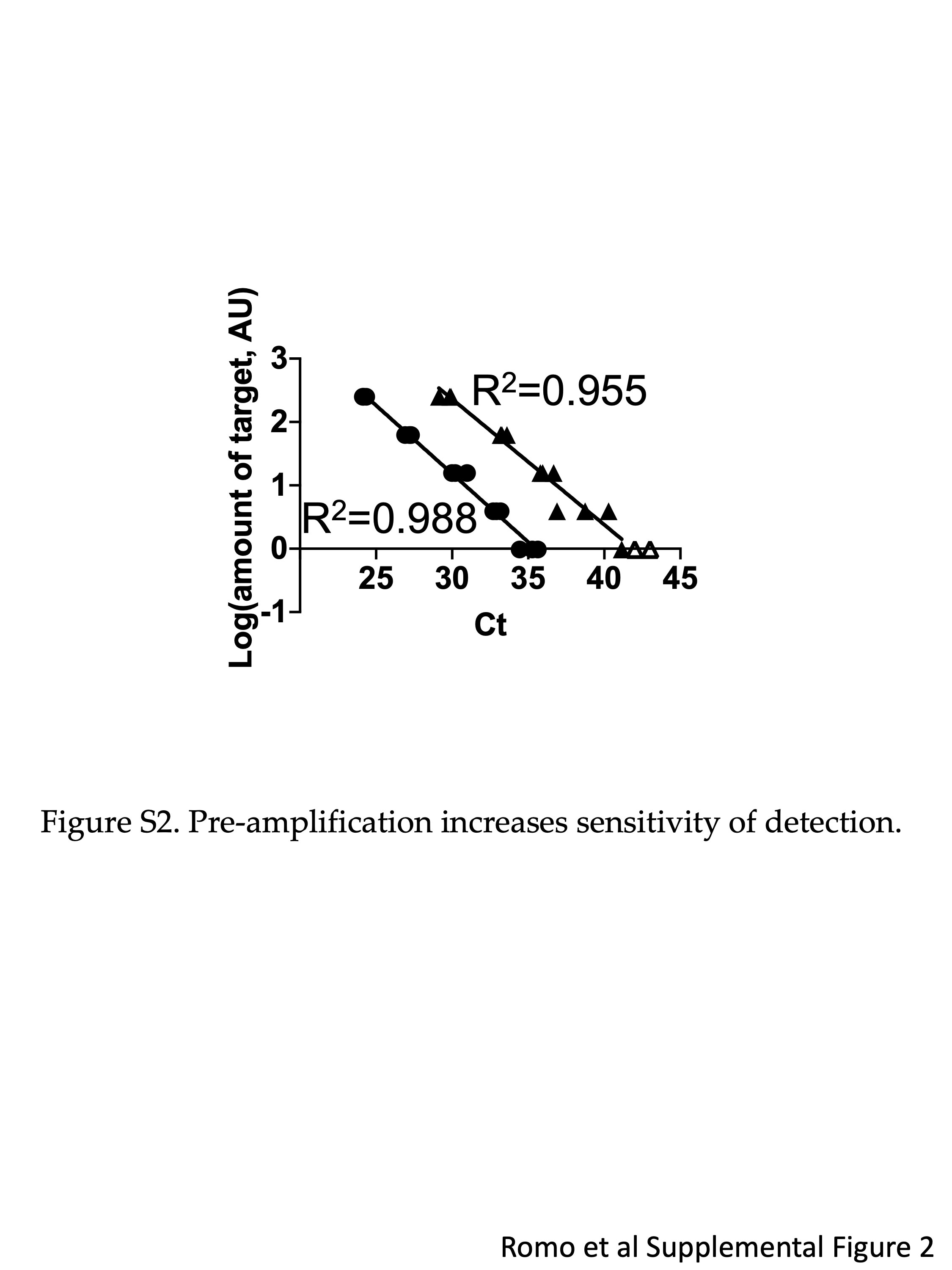

Supplement: Supplementary file 1 [file jof-06-00100-s001.zip › Supplementary_Materials/Supplemental_Figures_JPEG/Fig-S2.jpg]
